# Understanding and exploiting the fatty acid desaturation system in *Rhodotorula toruloides*

**DOI:** 10.1186/s13068-021-01924-y

**Published:** 2021-03-19

**Authors:** Yanbin Liu, Chong Mei John Koh, Sihui Amy Yap, Lin Cai, Lianghui Ji

**Affiliations:** 1 Temasek Life Sciences Laboratory, 1 Research Link, National University of Singapore, Singapore, 117604 Singapore; 2 School of Biological Sciences, Nanyang Technological University, 60 Nanyang Drive, Singapore, 637551 Singapore

**Keywords:** Fatty acid desaturase, Lipid, γ-Linolenic acid, Palmitoleic acid, Regulation

## Abstract

**Background:**

*Rhodotorula toruloides* is a robust producer of triacylglycerol owing to its fast growth rate and strong metabolic flux under conditions of high cell density fermentation. However, the molecular basis of fatty acid biosynthesis, desaturation and regulation remains elusive.

**Results:**

We present the molecular characterization of four fatty acid desaturase (FAD) genes in *R. toruloides*. Biosynthesis of oleic acid (OA) and palmitoleic acid (POA) was conferred by a single-copy ∆9 Fad (Ole1) as targeted deletion of which abolished the biosynthesis of all unsaturated fatty acids. Conversion of OA to linoleic acid (LA) and α-linolenic acid (ALA) was predominantly catalyzed by the bifunctional ∆12/∆15 Fad2. FAD4 was found to encode a trifunctional ∆9/∆12/∆15 FAD, playing important roles in lipid and biomass production as well as stress resistance. Furthermore, an abundantly transcribed *OLE1*-related gene, *OLE2* encoding a 149-aa protein, was shown to regulate Ole1 regioselectivity. Like other fungi, the transcription of FAD genes was controlled by nitrogen levels and fatty acids in the medium. A conserved DNA motif, (T/C)(G/A)TTGCAGA(T/C)CCCAG, was demonstrated to mediate the transcription of *OLE1* by POA/OA. The applications of these FAD genes were illustrated by engineering high-level production of OA and γ-linolenic acid (GLA).

**Conclusion:**

Our work has gained novel insights on the transcriptional regulation of FAD genes, evolution of FAD enzymes and their roles in UFA biosynthesis, membrane stress resistance and, cell mass and total fatty acid production. Our findings should illuminate fatty acid metabolic engineering in *R. toruloides* and beyond.

**Supplementary Information:**

The online version contains supplementary material available at 10.1186/s13068-021-01924-y.

## Background

Unsaturated fatty acids (UFAs) are fatty acids containing one (mono-unsaturated fatty acid, MUFAs) or more double bonds (polyunsaturated fatty acids, PUFAs) in various positions and configurations in the carbon backbone. UFAs play important roles in membrane fluidity and serve as precursors for the biosynthesis of many bioactive molecules, such as lipid mediators, pheromones, eicosanoids and growth regulators [1].

Fatty acid desaturases (FADs) catalyze the sequential desaturation of fatty acids, leading to the production of MUFAs and PUFAs. FADs are classified into two groups, water-soluble acyl–acyl carrier protein (ACP) desaturases restricted in plant plastid [2, 3] and integral membrane type FADs, which share the highly conserved membrane-spanning motif, H(X)_3-4_H(X)_7-41_H(X)_2-3_HH(X)_61-189_H(X)_2-3_HH [4]. FADs can also be functionally categorized as front-end and methyl-end desaturase, which introduces a double bond towards the carboxyl terminus and methyl-end of the fatty acyl chain, respectively [3]. The front-end desaturases, such as Δ4, Δ5, Δ6 and Δ8 FADs, contain a specific N-terminal cytochrome b5-like domain, and are generally found in animals and lower eukaryotic microorganisms [5, 6].

The expression of FADs is usually regulated by fatty acids, nutrient and environmental cues. For example*, Saccharomyces cerevisiae OLE1* expression is regulated at transcriptional and post-transcriptional (mRNA or protein stability) levels: the transcription is activated by unsaturated fatty acids through ubiquitin-mediated proteolytic processing of two membrane proteins, Mga2p and Spt23p, triggering their nuclear targeting to become transcriptional co-activators. Mga2p is believed to be a sensor for unsaturated fatty acid and it also regulates *OLE1* transcripts stability by modulating exonuclease activity [7, 8]. These regulatory mechanisms appear to be quite conserved among different fungi [9].

Different fatty acids vary greatly in nutritional value and biological functions due to their unique fatty acid configuration in triacylglycerol, degree of desaturation and chain length. For example, γ-linolenic acid (GLA, 18:3Δ^6,9,12^) has anti-inflammation property and has applications in the treatment for atopic eczema, diabetes, heart disease, high blood pressure, arthritis, Alzheimer's disease, etc. [10, 11]. GLA is present in relatively low levels in oils extracted from a small number of plant seeds, such as those of evening primrose (*Oenothera biennis*) (8–10% of total fatty acid), blackcurrant (15–20%) and *Borago officinalis* (~ 20%) [12]. Although filamentous fungi such as *Cunninghamella echinulata* [13] and *Mortierella isabellina* [14] also produce GLA, they are not ideal hosts for industrial production due to slow growth, low lipid content and high viscosity during fermentation.

*R. toruloides* is an oleaginous yeast producing low levels of PUFAs, including linoleic acid (LA, 18:2Δ^9,12^) and α-linolenic acid (ALA, 18:3Δ^9,12,15^) [15]. Metabolic engineering offers an opportunity to drastically change its fatty acid composition and productivity [16–20]. As a highly robust oil producer, *R. toruloides* is a potentially powerful platform for fatty acid engineering and production [21–24]. *R. toruloides* remains a challenging host to work with due to its highly GC-rich genome (~ 62%); unusual regulation of gene expression and limited engineering tools [25–27]. To date, two *Rhodotorula* FAD genes have been reported, a stearoyl-CoA desaturase gene from *R. toruloides* IFFO 0880 [28] and a Δ12/Δ15 bifunctional desaturase gene from *R. kratochvilovae* YM25235 [29]. To further facilitate PUFA metabolic engineering in *R. toruloides*, we characterized four FADs identified, analyzing their gene/protein organizations, transcriptional regulations and effects of gene deletion and overexpression in the native host on fatty acid biosynthesis, stress responses and cell mass production. We illustrated multi-step metabolic engineering routes, via loss-of-function and gain-of-function approaches, for the efficient production of high-value fatty acids in *R. toruloides*.

## Results and discussion

### Identification of fatty acid desaturase genes in *R. toruloides*

To identify FAD genes, several well-studied enzymes were used as the queries for tBLASTn search (NCBI, USA) against the genomes of *R. toruloides* strain ATCC 10657 and 204091. Using *S. cerevisiae* Δ9 stearoyl-CoA desaturase (GenBank accession no. CAA96757, ScOle1p), *Mortierella alpina* Δ12 FAD (ADE06660, MaFAD2), *M. alpina* Δ6 FAD (AAL73949, MaFAD6) and *Euglena gracilis* Δ8 FAD (ADD51570) as queries, we identified 3 homologous genes, which were tentatively named *OLE1*, *FAD2* and *FAD4*. However, homolog of *M. alpina* Δ5 FAD (ACM89303), *Thraustochytrium sp.* Δ4 FAD (AAM09688) and *Saprolegnia diclina* Δ17 FAD (AY373823) was not found.

Gene organization of FADs, such as coding sequence (CDS), 5′ and 3′UTR (untranslated region), was determined by incorporating the sequences of 5′ and 3′ RACE (rapid amplification of cDNA ends), RT-PCR and whole transcriptome. *OLE1*, *FAD2* and *FAD4* contain 7, 4 and 4 exons, encoding 545, 451 and 609 aa, respectively (Table 1 and Additional file 1: Fig. S1a). All splicing junctions abide strictly to the canonical GU-AG rule. Notably, *OLE1* and *FAD2* transcripts have long 3′UTRs, 296 nt and 261 nt, respectively, while *FAD4* has a long 5′UTR of 349 nt (Table 1 and Additional file 1: Fig. S1a).

**Table 1:** Gene annotations

Gene	CDS length (nt)	Scaffold No	5′UTR (nt)	3′UTR (nt)	Exon	Protein (aa)	Best hit (identity)^a^
*OLE1*	2304	9	160	296	7	545	XP_016270987.1 (97%)
*FAD2*	1703	24	21	261	4	451	XP_016269356.1 (97%)
*FAD4*	1604	25	349	69	4	476	XP_016270876.1 (94%)
*OLE2*	848	9	NA^b^	NA	5^b^	149	XP_016270986 (67%)

^**a**^The GenBank accession numbers of the best hits in *R. toruloides* NP11. The number in parenthesis indicates the sequence identity of the encoded protein

^**b**^Not available. Exons and CDS were predicted according to the annotation of ATCC 204091

Ole1 was predicted to contain two transmembrane helices while Fad2 and Fad4 have three (Additional file 1: Fig. S1b). All three FADs contain the pfam00487 membrane domain that is highly conserved in different organisms [30], and other FAD-signature motifs, such as cd03505 (Δ9 FAD-like), cd03507 (Δ12 FAD-like) or cd03506 (Δ6 FAD-like) [31]. Surprisingly, only Ole1 contains the fungus-specific fused cytochrome *b5* heme/steroid binding domain (pfam00173) at the carboxyl terminus (Additional file 1: Fig. S1b), suggesting Fad2 and Fad4 rely on the free form cytochrome *b5* reductase to couple the fatty acid desaturation reaction. Like many reported membrane-bound FADs, all three FADs contain three conserved histidine boxes, H(X)_3-4_H, H(X)_2-3_HH and H/Q(X)_2-3_HH (Additional file 1: Fig. S1b and Additional file 1: Fig. S2), which form the di-iron complex that is essential for the desaturation reaction. The positions of the first two histidine boxes are highly conserved, separated by 31–32 aa. The 3rd histidine box is located 130 aa from the 2nd one in Ole1 while the spacing in Fad2 and Fad4 is 185 aa and 193 aa, respectively (Additional file 1: Fig. S2). Notably, the 3rd histidine box of Fad4 has an imperfect sequence, **Q**xx**HH**, which is often observed in the front-end desaturases [32].

Phylogenetic analysis of eukaryotic FADs showed that *R. toruloides* homologs fell into three distinct groups (Additional file 1: Fig. S3). Consistent with previous work [3], it is difficult to distinguish mono-functional Δ12 FAD and Δ15 FAD from bifunctional Δ12/Δ15 FAD based on the amino acid sequences. Bifunctional enzymes with both Δ12 and Δ15 regioselectivity are believed to derive from Δ12 FAD [3]. In nature, *R. toruloides* strains are usually haploids with two mating types (*A1* and *A2*) [33]. Sequence comparison revealed no amino acid sequence difference in strains of the same mating type (mating type *A1* strains ATCC 10657, ATCC 204091 and IFFO0880 or mating type *A2* strains ATCC 10788, MTCC457 and CECT 1137) [34] while 94.3 – 97.2% identities were observed between different mating types (Additional file 1: Table S1). However, the nucleotide sequence identities were much lower, ranging from 87.1 to 88.9%.

### Regulation of FAD gene transcription

**Fig. 1:**
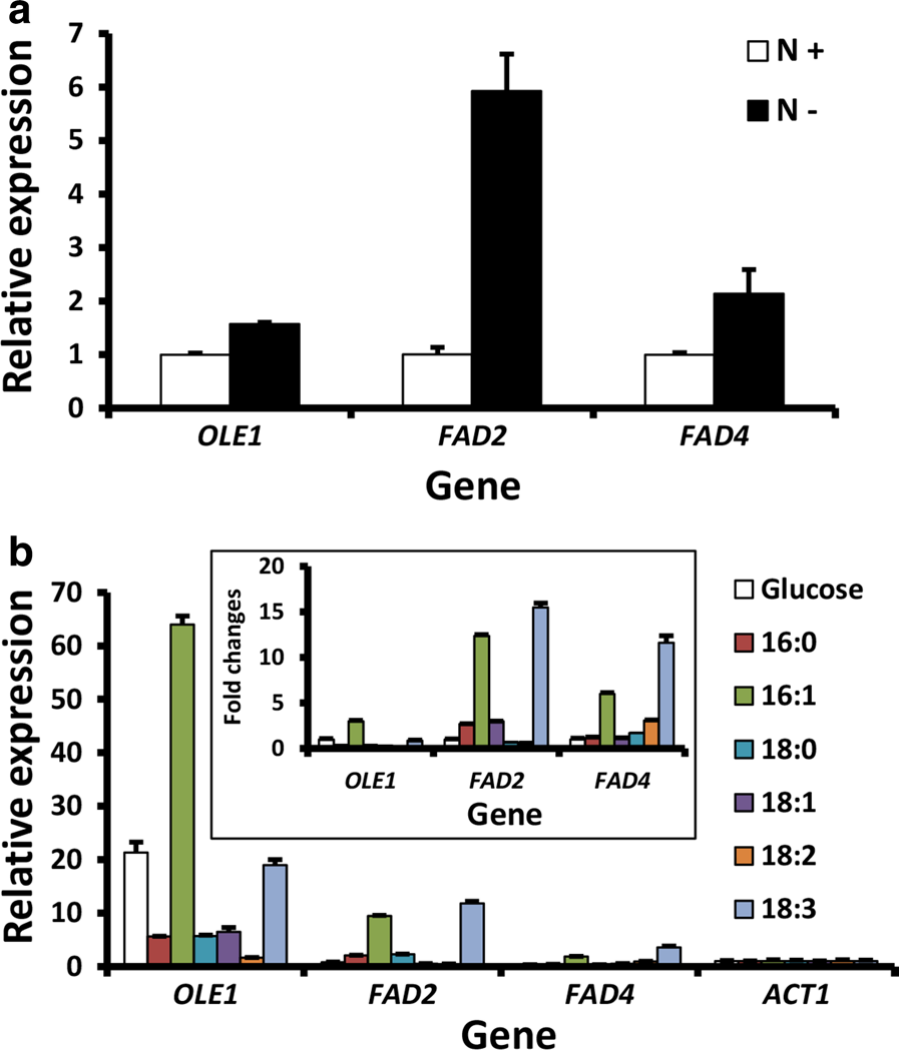
qRT-PCR analysis of FAD genes. **a** Effect of nitrogen starvation on FAD gene transcription. *R. toruloides* seed culture established in YPD broth was washed in water, inoculated to yeast nitrogen base (YNB) or YNB-N^−^ (without amino acid supplement and ammonium sulfate) and cultured at 28 °C. Cells for total RNA extraction were sampled at 12 and 24 h after inoculation. The relative mRNA levels were calculated by 2-ΔΔCt method and represented as the ratio between the two media. **b** Effects of exogenous fatty acid supplementation on transcription of FADs. The 2-ΔCt method was applied for the data analysis and represented as relative expression against the reference gene. The inlet shows the levels of each mRNA from cells cultured in different media after normalizing against that in the control medium (glucose as the carbon source). Seed culture was washed with water twice and inoculated in carbon source-free YNB broth, which was individually supplemented with different fatty acids (10 g/L) and cultured at 28 °C for 8 h with 280 rpm agitation. Glucose (10 g/L) was used as the control, and Tergitol NP40 was supplemented at 1% (w/v) to facilitate fatty acid absorption in all treatments. For qPCR analysis, actin encoding gene (*ACT1*) was used as the reference and error bars represent the standard derivations of triplicates

Fatty acid biosynthesis and lipid accumulation are often regulated by environmental and nutrient cues [35]. Indeed, qRT-PCR analysis showed that *OLE1*, *FAD2* and *FAD4* mRNA levels were significantly increased under 6-h nitrogen starvation, and the level of *OLE1*, *FAD2* and *FAD4* transcripts in nitrogen-free YNB medium was 1.6, 5.9- and 2.1-fold higher than in YNB medium, respectively (Fig. 1a). These suggest the involvement of common nitrogen-regulated transcriptional factors.

Studies on the gene transcriptional effects of exogenous fatty acids were concentrated on Δ9 FAD. Strong repressive effects of UFAs were reported in several yeasts [36–38], however, different effects were also reported, with minor repression or no effect in other yeasts [39, 40]. With regard to *FAD2* and *FAD4*, it is interesting to note that UFAs resulted in different regulatory patterns [41–43]. Thus, *R. toruloides* FADs were investigated on their responses to different fatty acids as the sole carbon source. The three FAD genes showed significantly different transcription levels (Fig. 1b). *OLE1* mRNA was the most abundant, the transcription of which could be strongly induced by palmitoleic acid (POA, 16:1^∆9^) and significantly depressed by most other fatty acids (Fig. 1b). *FAD2* transcription was significantly induced by most fatty acids tested except 18:2. Like *OLE1*, POA was the strongest inducer for *FAD2*. This regulatory pattern was similar to the OA-inducible pattern of *Yarrowia lipolytica* ∆12 FAD [44]. *FAD4* transcription, on the other hand, was significantly induced by POA, 18:2 and 18:3 although the overall mRNA level was the lowest among the three FAD transcripts. Taken together, the general regulatory network of FAD gene transcription is quite conserved among different fungi. Nevertheless, *R. toruloides* has evolved distinct regulatory controls, such as the strong induction of FAD gene expression by POA. It would be interesting to see how common this phenomenon is in other microbes although, as suggested previously [45], it could result from its unique evolutionary history and specific niche it resides. POA is a rare fatty acid present in the cells at very low levels in most systems. We speculate that supplying high levels of this may drastically change the membrane structure or fluidity, resulting in a stress response in the cells.

### Molecular basis of transcriptional control of FAD genes

**Fig. 2:**
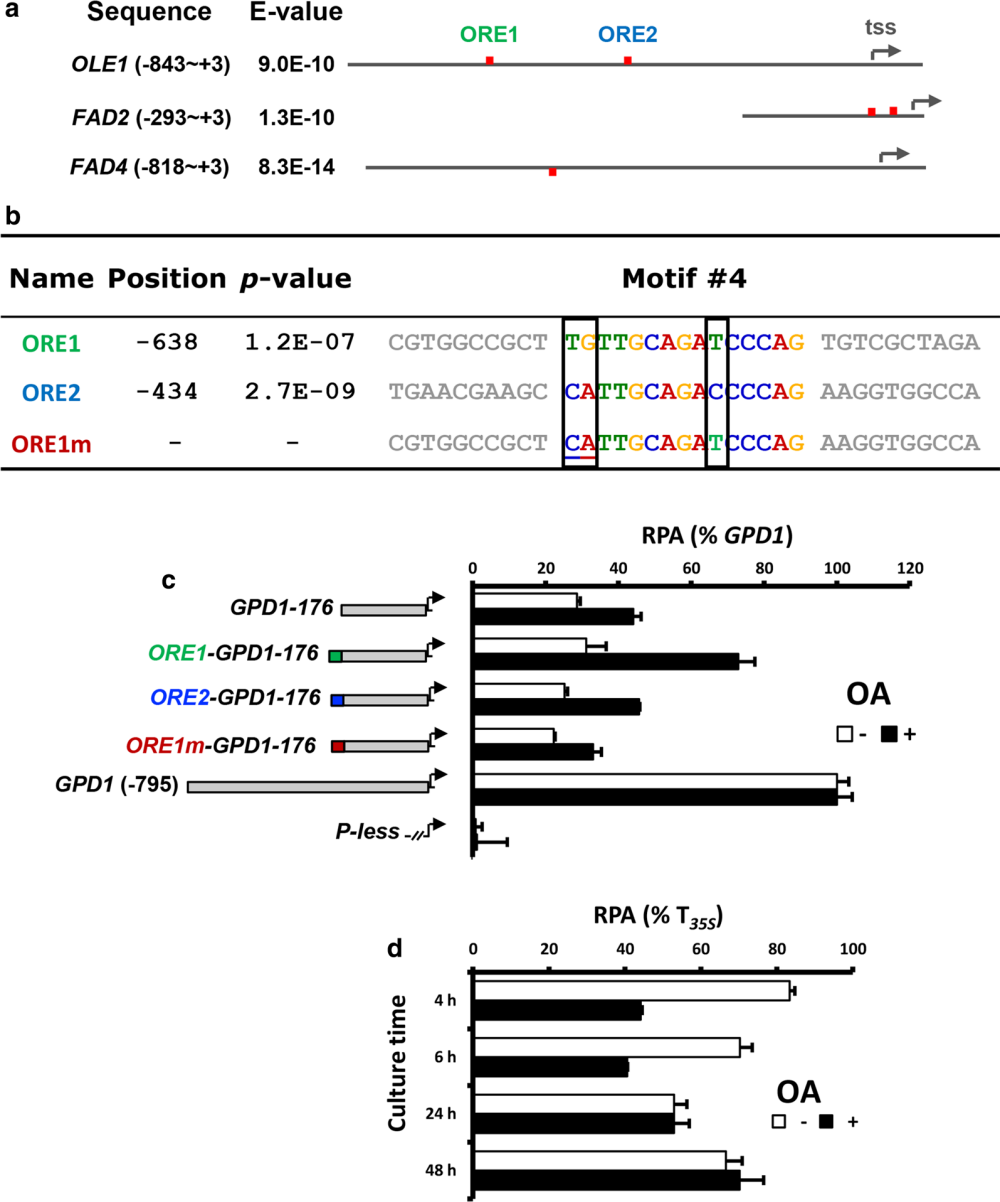
Identification of putative transcriptional regulatory elements. **a** Distribution of putative fatty acid responsive elements in the upstream sequences of 3 FAD genes. Common DNA motifs were identified using the MEME suite at http://meme-suite.org. *E*-value is the estimate of the number of motifs expected to find by chance if the letters in the input sequences were shuffled. tss: transcription start site. **b** Sequence alignment of two candidate *OLE1* Regulatory Element (ORE) in the *OLE1* promoter. ORE1m is the artificially generated mutant of ORE1, containing 2-nt substitutions. **c** Luciferase gene reporter assay of hybrid *GPD1* promoters. The promoter structures are illustrated on the left, where green, blue and red bars show the location of the inserted DNA motifs. **d** Effect of *OLE1* terminator on gene expression. The *Cauliflower mosaic virus* 35S gene terminator in P_*OLE1*_*::*Rt*LUC2::*T_*35S*_ was replaced with the 328 bp terminator of *OLE1*. The relative promoter activity (RPA) was calculated by normalizing the value against the reading of control construct (P_*OLE1*_*::*Rt*LUC2::*T_35S_) of the same culture conditions and sampling time point

To investigate the transcriptional regulations, the upstream sequence of *OLE1* (-843 to -1 from the 1^st^ ATG codon) was cloned and analyzed by luciferase gene reporter assay. Time course study showed that the promoter was strongly induced by POA (peaked by 16.4-fold at 1 h) (Additional file 1: Fig. S4), which agrees well with the qRT-PCR results (Fig. 2b). The promoter was also induced by OA (peaked by 5.9-fold at 4 h). As POA is much more costly, OA was used as the inducer in later transcriptional studies.

To identify the common *cis*-acting elements involved in the transcriptional regulation by fatty acids, the upstream sequences of *OLE1*, *FAD2* and *FAD4* (Additional file 1) were analyzed using the MEME suite [46], leading to the identification of a 15-nt conserved DNA motif (Fig. 2a). In *OLE1*, two such motifs with 3-nt variations were found, at -638 and -434 from the translational start site. The motifs were tentatively named ORE1 and ORE2 (OLE1 Regulatory Element), respectively, Fig. 2b). To confirm its function, ORE1 and ORE2 was individually fused to the 5′ end of the basal *GPD1-176* promoter (-176 to the 1^st^ ATG codon) [25]. Neither ORE1 nor ORE2 significantly affected *GPD1-176* activity when the reporter strains were cultured in YPD medium (Fig. 2c). In contrast, the ORE1-*GPD1-176* promoter showed 1.5-fold higher activity than *GPD1-176* when cultured in OA-supplemented medium, whereas ORE2 showed negligible effect. To determine which of the three substituted nucleotides was functionally important, an ORE1 mutant (ORE1m) was created by converting its first 2 nucleotides to the corresponding residues in ORE2 (Fig. 2b). Reporter assay revealed a complete loss of oleate-inducing effect after the sequence change (Fig. 2c). This suggests that ORE1, and possibly some related motifs, plays a significant role in regulating FAD gene transcription. The data also suggest the possibility to engineer a strong OA/POA-inducible gene expression system in *R. toruloides* by using the *OLE1* promoter and ORE1 motif.

The transcripts of *OLE1* and *FAD2* have long 3′UTRs (Table 1 and Additional file 1: Fig. S1a). To investigate if the 3′UTR of *OLE1* has any role in regulating *OLE1* expression, the luciferase reporter construct was modified by replacing the terminator of *Cauliflower mosaic virus* (CaMV) 35S gene with that of *OLE1*, including the entire 296-bp 3′ UTR and 32-bp downstream sequence (Additional file 1). This resulted in a significant drop in the luciferase activity (Fig. 2d). This explains the discrepancy between the results of qRT-PCR (Fig. 1b) and promoter reporter assay (Additional file 1: Fig. S4). Thus, OA and POA modulate *OLE1* transcription via the *cis*-acting elements located in both the upstream and downstream regions of the gene.

### *OLE1* is essential for cell viability and biosynthesis of oleic acid and palmitoleic acid

We reported previously that gene deletion frequency could reach more than 95% when using the *KU70* knockout mutant [47]. However, no *OLE1* deletion mutant was obtained after repeated attempts, regardless of OA supplementation to culture media. Subsequently, a true deletion mutant (*ole1Δ*) was generated in another strain, *R. toruloides* C3 (Additional file 1: Fig. S5a). Sequence analysis revealed that the single-copy *OLE1* gene is highly conserved between C3 and ATCC 10657 strains, with only 7-nt substitutions that occurred in intron regions.

**Fig. 3:**
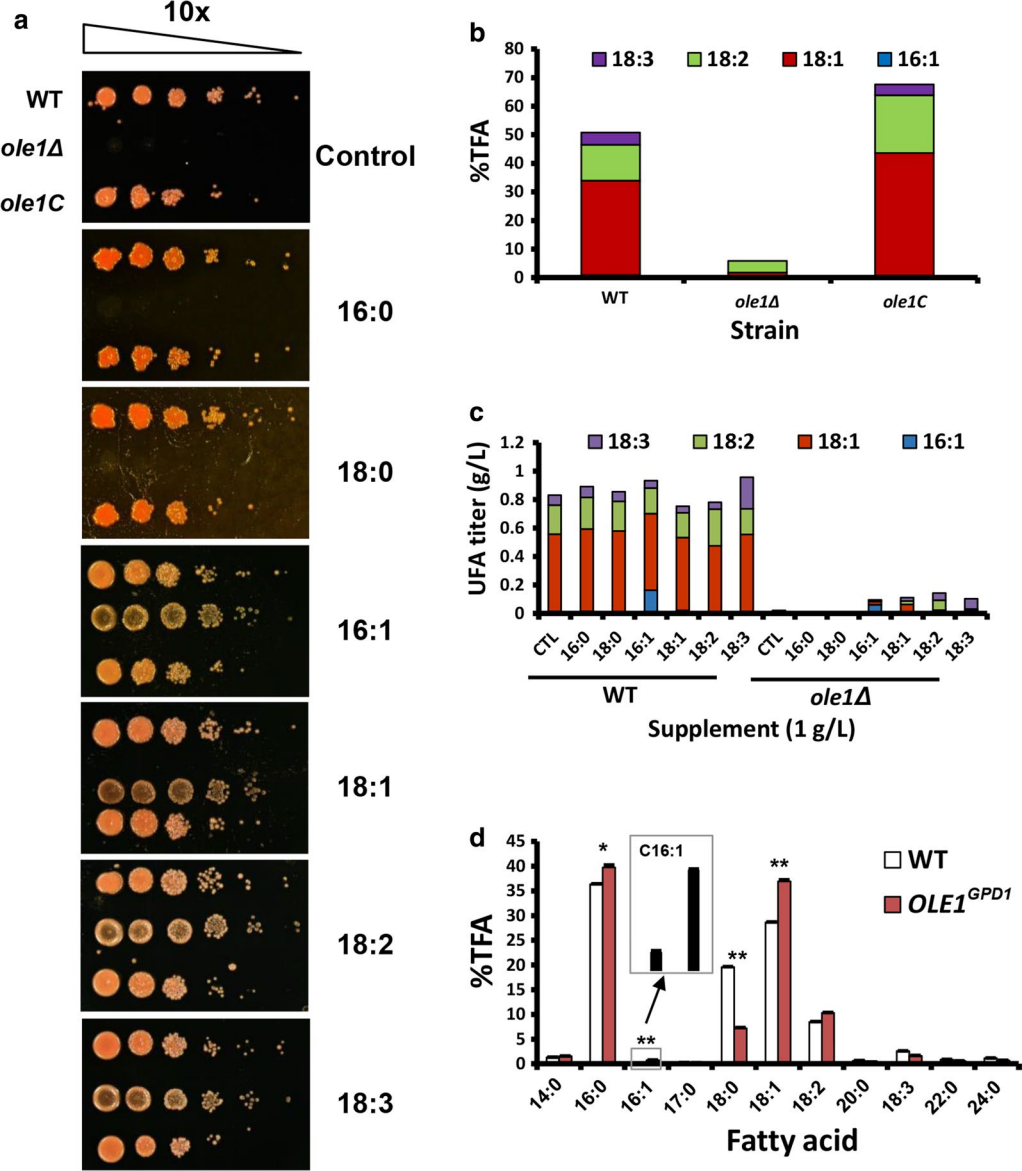
Functional characterization of *OLE1*. **a** Genetic and chemical complementation of *ole1Δ*. *ole1C* strain is an *ole1Δ* derivative containing a randomly inserted construct comprising 641-bp *OLE1* promoter, full-length *OLE1* cDNA and 328-bp *OLE1* terminator derived from ATCC 10,657. Cells were cultured on YPD agar in the presence ( +) or absence (−) of various fatty acids at 1% w/v. **b** Unsaturated fatty acid profiles in *R. toruloides* wild-type (WT), *ole1Δ* and *ole1C* strains. Strains were cultured in YPD broth or YPDtO broth (YPD broth supplemented with 0.1% w/v OA and 0.5% w/v Tergitol NP40) until exponential phase. Water-washed (2 times) cells were inoculated to GJm3 medium and cultured for 5 days. **c** Fatty acid profiles of *R. toruloides* WT and *ole1Δ* mutant. Cells were cultured in GJm3 medium supplemented with different fatty acids (0.1%, w/v) for 4 days. CTL represents the cells cultured in GJm3 medium in the absence of any fatty acid. (d) Fatty acid profiles in wild-type strain (WT) and *OLE1* overexpression mutant (*OLE1*^*GPD1*^). Both strains were cultured in GJm3 medium for 5 days. %TFA represents the weight percentage of total fatty acid. Error bars represent the standard derivations of triplicates. Student’s *t*-test was used for statistical analysis, where possibility less than 0.05% and 0.01% was marked as * and **, respectively

As expected, *ole1Δ* was unable to grow in medium with glucose or saturated fatty acids as the sole carbon source (Fig. 3a). In contrast, supplementation of any UFAs, such as 16:1, 18:1, 18:2 or 18:3, rescued the growth defect caused by the lack of *OLE1* gene (Fig. 3a). Therefore, *ole1Δ* is an UFA-auxotrophic mutant, resembling its counterpart in *S. cerevisiae* [48]. *ole1Δ* was inactive in UFA biosynthesis (Fig. 3b). The small amount of 18:1 detected was probably derived from the inoculant cells that had been cultured in OA-supplemented medium (Fig. 3b). As expected, re-introduction of wild-type *OLE1* gene into the *ole1Δ* genome completely restored the growth defects (Fig. 3a) and lipid biosynthesis (Fig. 3b). Furthermore, UFA, such as 16:1, 18:1, 18:2 and 18:3, partially restored the fatty acid profile of *ole1Δ* although fatty acid titer remained much lower due to the defect in cell growth (Fig. 3c). Supplement of 16:1 or 18:1, but not 16:0 or 18:0, was able to complement the growth and fatty acid biosynthesis defects of *ole1Δ* (Fig. 3a and c)*.* These suggest that other FADs were functional in the absence of Ole1. Overexpressing *OLE1* using the strong *GPD1* promoter resulted in a 5.3- and 1.3-fold increase in 16:1 and 18:1 level, respectively (Fig. 3d). Collectively, our results suggest that, similar to its homologs in *S. cerevisiae* and *Y. lipolytica* [49], Ole1 is a Δ9 desaturase with a strong substrate preference to stearoyl-CoA over palmitoyl-CoA. Our data also support the previous studies that oleic acid plays a central role in fungal growth and metabolism [4, 48, 50]. To date, Δ9 FAD null mutants have been reported only in two ascomycetous yeasts, *S. cerevisiae* and *Candida parapsilosis* [4, 51]. To the best of our knowledge, this is the first report on the phenotypes of *OLE1* null mutant in basidiomycetous fungi and oleaginous yeasts.

### *FAD2 *encodes a Δ12/Δ15 bifunctional fatty acid desaturase

**Fig. 4:**
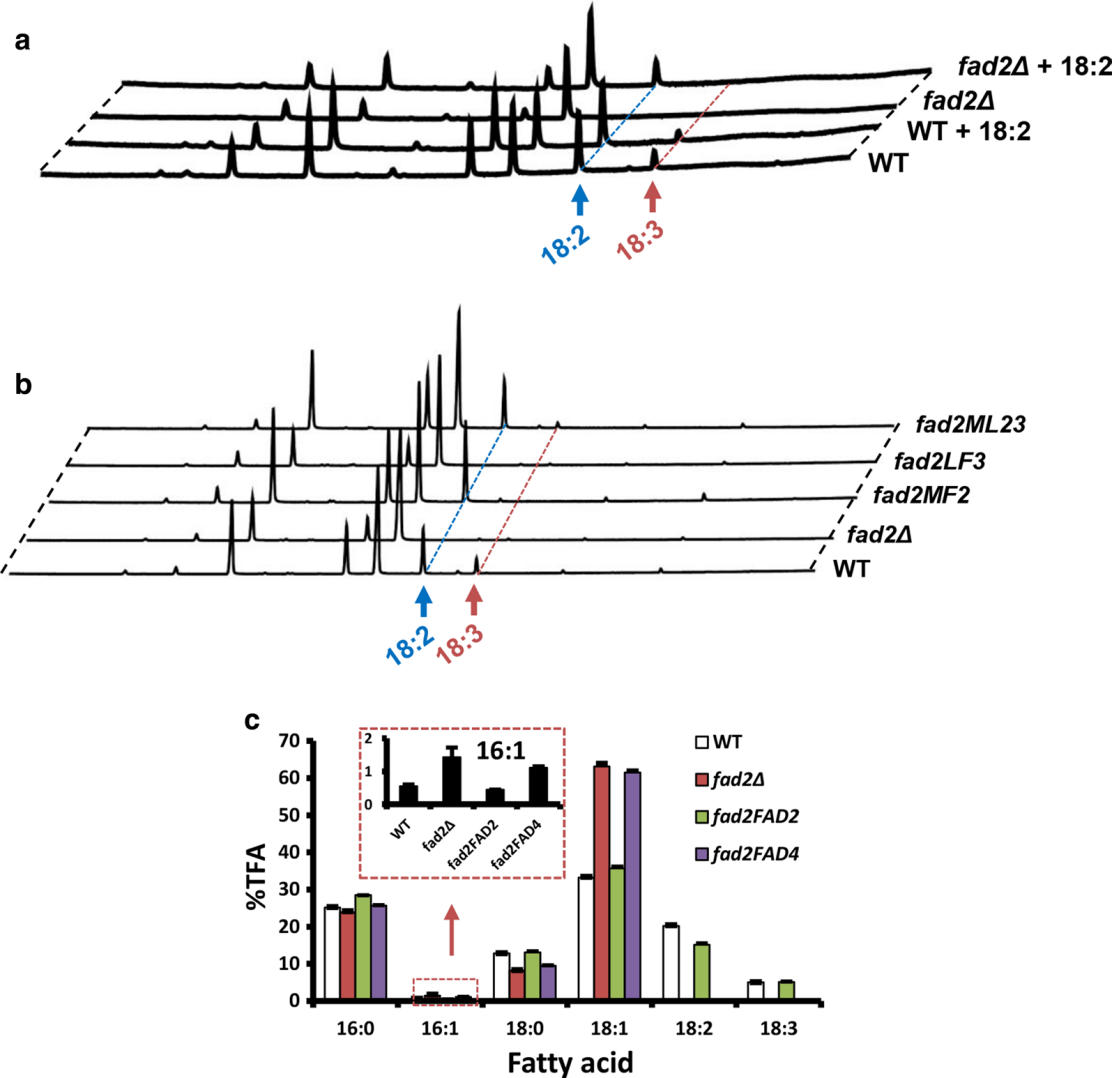
Characterization of *FAD2*. **a** GCMS chromatographs of FAMEs from WT and *FAD2* deletion mutant (fad2Δ). + 18:2 indicates cells cultured in medium supplemented with 18:2. **b** Complementation of fatty acid production defect of *fad2Δ* by overexpression of known heterologous Δ12- and/or Δ15-fatty acid desaturase. *WT* wild-type strain, *fad2MF2* fad2Δ expressing the codon-optimized gene encoding *M. alpina* Δ12 FAD (Ma*FAD2-2*), *fad2LF3* fad2Δ expressing the codon-optimized flax Δ15 FAD (Lu*FAD3-2*), *fad2ML* fad2Δ expressing both Ma*FAD2-2* and Lu*FAD3-2*. **c** Fatty acid profiles of WT, fad2Δ, fad2FAD2 and fad2FAD4. %TFA represents weight percentage of total fatty acids. Error bars represent standard derivations of triplicates

The *FAD2* null mutant (*fad2Δ*, Table 2) was also generated (Additional file 1: Fig. S5b). Unlike *ole1Δ*, *fad2Δ* was able to grow normally without the presence of 18:2 in media (Fig. 6a, b), suggesting that Fad2 is dispensable for cell growth. The production of both 18:2 and 18:3 was abolished in *fad2Δ* while 18:1 was normal (Fig. 4a). These data suggest that Fad2 functions as both the Δ12 and Δ15 FAD to convert 18:1 to 18:2 and 18:3.

**Table 2:** Strains and plasmids used in this study

Strain/plasmid	Characteristics	Source
Strains		
* R. toruloides* ATCC 10657	*R. toruloides* host	ATCC
C3	A haploid strain isolated from Singapore	This study
* ole1Δ*	*OLE1*::*Hyg*^*R*^ in C3 background	This study
* R. toruloides* Rt1ck	Δku70e, *ku70Δ::loxP*^a^, marker free, designed as wild-type strain	[47]
* OLE1*^*GPD1*^	*ku70Δ::loxP car2Δ::P*_*GPD1*_*-OLE1-Hyg*^*R*b^	This report
* fad2Δ*	*ku70Δ::loxP fad2Δ::Hyg*^*R*^	This report
* fad2e*	*ku70Δ::loxP fad2Δ::loxP*	This report
* fad2C*	*ku70Δ::loxP fad4Δ::loxP car2Δ::P*_*GPD1*_*-FAD2-Hyg*^*R*^* (alias fad2FAD2)*	This report
* fad4Δ*	*ku70Δ::loxP fad4Δ::Hyg*^*R*^	This report
* fad4e*	*ku70Δ::loxP fad4Δ::loxP*	This report
* fad4FAD4*	*ku70Δ::loxP fad4Δ::loxP car2Δ::P*_*GPD1*_*-FAD4-Hyg*^*R*^* (alias fad4FAD4)*	This report
* fad4FAD4a*	*ku70Δ::loxP fad4Δ::loxP car2Δ::FAD4 allele-Hyg*^*R*^	This report
* fad24Δ*	*ku70Δ::loxP fad4::loxP fad2Δ::Hyg*^*R*^	This report
* fad24e*	*ku70Δ::loxP fad4Δ::loxP fad2Δ::loxP*	This report
* fad2FAD4*	*ku70Δ::loxP fad2Δ::loxP car2Δ::P*_*GPD1*_*-FAD4-Hyg*^*R*^	This report
* fad4FAD2*	*ku70Δ::loxP fad4Δ::loxP car2Δ::P*_*GPD1*_*-FAD2-Hyg*^*R*^	This report
* fad24FAD2*	*ku70Δ::loxP fad4Δ::loxP fad2Δ::loxP car2Δ::P*_*GPD1*_*-FAD2-Hyg*^*R*^	This report
* fad24FAD4*	*ku70Δ::loxP fad4Δ::loxP fad2Δ::loxP car2Δ::P*_*GPD1*_*-FAD4-Hyg*^*R*^	This report
* fad2MF2*	*ku70Δ::loxP fad2Δ::loxP car2Δ::P*_*GPD1*_*-MaFAD2-2-Hyg*^*R*^	This report
* fad2LF3*	*ku70Δ::loxP fad2Δ::loxP car2Δ::P*_*GPD1*_*-LuFAD3-2-Hyg*^*R*^	This report
* fad2ML*	*ku70Δ::loxP fad2Δ::loxP car2Δ::P*_*GPD1*_*-MaFAD2-2-P*_*GPD1*_*-LuFAD3-2-Hyg*^*R*^	This report
* fad2OLE1*	*ku70Δ::loxP fad2Δ::loxP car2Δ::P*_*LPD1in*_*-OLE1-Hyg*^*R*^	This report
* fad24OLE1*	*ku70Δ::loxP fad4Δ::loxP fad2Δ::loxP car2Δ::P*_*LPD1in*_*-OLE1-Hyg*^*R*^	This report
* fad2MF26*	*ku70Δ::loxP fad2Δ::loxP car2Δ::P*_*LDP1in*_*-MaFAD2-2-P*_*LDP1in*_*-MaFAD6-2-Hyg*^*R*^	This report
* fad24MF26*	*ku70Δ::loxP fad2Δ::loxP fad4Δ::loxP car2Δ::P*_*LDP1in*_*-MaFAD2-2-P*_*LDP1in*_*-MaFAD6-2-Hyg*^*R*^	This report
* A. tumefaciens* AGL1	*Agrobacterium* host for ATMT	[71]
* E. coli* XL1-Blue	*recA1 endA1 gyrA96 thi-1 hsdR17 supE44 relA1 lac*, *E. coli* host for routine DNA manipulation	Stratagene, USA
Plasmids		
pEX2	*Sp*^*R*^^c^, binary vector pZP200 derivative	[69]
pKC2	*Sp*^*R*^, pEX2 derivative, *CAR2L-P*_*GPD1*_*-RtGFP-Hyg*^*R*^*-CAR2R*^d^, for promoter analysis, gene expression and CAR2 locus integration	[68]
pKCL2	*Sp*^*R*^, pKCL2 derivative, *CAR2L-P*_*GPD1*_*-RtLUC2-Hyg*^*R*^*-CAR2R*, for promoter analysis, gene expression and CAR2 locus integration	[68]
pKCL25	*Sp*^*R*^, pKCL2 derivative, *CAR2L-P*_*GPD1-176*_*-RtLUC2-Hyg*^*R*^*-CAR2R*, for promoter analysis and CAR2 locus integration	This report
pKCL254	*Sp*^*R*^, pKCL2 derivative, *CAR2L-ORE1-P*_*GPD1-176*_*-RtLUC2-35T-Hyg*^*R*^*-CAR2R*, for promoter reporter assay and CAR2 locus integration	This report
pKCL255	*Sp*^*R*^, pKCL2 derivative, *CAR2L-ORE2-P*_*GPD1-176*_*-RtLUC2-35T-Hyg*^*R*^*-CAR2R*, for promoter reporter assay and CAR2 locus integration	This report
pKCL256	*Sp*^*R*^, pKCL2 derivative, *CAR2L-ORE1m-P*_*GPD1-176*_*-RtLUC2-35T-Hyg*^*R*^*-CAR2R*, for promoter reporter assay and CAR2 locus integration	This report
pKCLF66	*Sp*^*R*^, pKCL2 derivative, *CAR2L-P*_*OLE1-641*_*-RtLUC2-35T-Hyg*^*R*^*-CAR2R*, for promoter reporter assay and CAR2 locus integration	This report
pKCLF661	*Sp*^*R*^, pKCL2 derivative, *CAR2L-P*_*OLE1-641*_*-RtLUC2-T*_*OLE1*_*-Hyg*^*R*^*-CAR2R*, for promoter reporter assay and CAR2 locus integration	This report
pKCLP4	*Sp*^*R*^, pKCL2 derivative, *CAR2L-P*_*LDP1in*_*-RtLUC2-Hyg*^*R*^*-CAR2R*, for *LDP1in* promoter driving gene overexpression and CAR2 locus integration	[26]
pKOFAD2	*Sp*^*R*^, pEX2 derivative, *FAD2L-Hyg*^*R*^-*FAD2R*, for deletion of *FAD2*	This report
pKOFAD4	*Sp*^*R*^, pEX2 derivative, *FAD4L-Hyg*^*R*^-*FAD4R*, for deletion of *FAD4*	This report
pNE1OLE1ca	*Sp*^*R*^, *CAR2L-OLE1 allele-Hyg*^*R*^*-CAR2R*, for complementation of *ole1Δ*	This report
pKC2FAD4	*Sp*^*R*^, *CAR2L-P*_*GPD1*_*-FAD4-Hyg*^*R*^*-CAR2R*, for overexpression of *FAD4*	This report
pKC2FAD4a	*Sp*^*R*^, *pKC2 derivative, Hyg*^*R*^*-FAD4 allele, for complementation*	This report
pKC2MF2	*Sp*^*R*^, *pKC2 derivative, CAR2L-P*_*GPD1*_*-MaFAD2-2-Hyg*^*R*^*-CAR2R*, for overexpression of Ma*FAD2-2*	This report
pKC2LF3	*Sp*^*R*^, *pKC2 derivative, CAR2L-P*_*GPD1*_*-LuFAD3-2-Hyg*^*R*^*-CAR2R*, for overexpression of Lu*FAD3-2*	This report
pKC2ML	*Sp*^*R*^, *pKC2 derivative, CAR2L-P*_*GPD1*_*-MaFAD2-2-P*_*GPD1*_*-LuFAD3-2-Hyg*^*R*^*-CAR2R*, for overexpression of Ma*FAD2-2* and Lu*FAD3-2*	This report
pKP4OLE1	*Sp*^*R*^*, pKCLP4 derivative, P*_*LDP1in*_*-RtLUC2-Hyg*^*R*^, for overexpression of *OLE1*	This report
pKP4MF2	*Sp*^*R*^, *pKCLP4derivative, CAR2L-P*_*LDP1in*_*-MaFAD2-2-Hyg*^*R*^*-CAR2R*, for overexpression of Ma*FAD2-2*	This report
pKP4MF6	*Sp*^*R*^, *pKCLP4 derivative, CAR2L-P*_*LDP1in*_*-*Ma*FAD6-2-Hyg*^*R*^*-CAR2R*, for overexpression of Ma*FAD6-2*	This report
pKP4MF26	*Sp*^*R*^, *pKCLP4 derivative, P*_*LDP1in*_*-MaFAD2-2-P*_*LDP1in*_*-* Ma*FAD6-2-Hyg*^*R*^, for overexpression of Mf*FAD2-2* and Mf*FAD6-2*	This report

^a^*loxP* (locus of X-over P1) used are the 34-bp lox77 and lox66 mutant Cre recombinase sites [72]

^b^*Hyg*^*R*^ represents the hygromycin resistance gene cassette P_*GPD1-3*_-*HPT-3*-T_*SV40*_, where P_*GPD1–3*_, *HPT-3* and T_*SV40*_ is the glyceraldehyde 3-phosphate dehydrogenase promoter of *Rhodotorula graminis* WP1 (JQ806386) [25], codon-optimized *E. coli* hygromycin phosphotransferase gene (JQ806387) [25] and the terminator of Simian virus 40 [73], respectively

^c^*Sp*^*R*^ represents the spectinomycin resistant gene

^d^T-DNA regions of the binary plasmids

The enzymatic function of Fad2 was further investigated by complement with certain substrates and genes. Addition of 18:2 did not restore the 18:3 level in *fad2Δ* (Fig. 4a), which is consistent with the lack of Δ15 FAD in *fad2Δ*. As *M. alpina* Δ12 FAD (MaFAD2) and *Linum usitatissimum* Δ15 FAD (LuFAD3) have been functionally confirmed [52, 53], the encoding genes were synthesized after codon optimization (Ma*FAD2-2* and Lu*FAD3-2*, respectively) and used in the test. Introduction of Ma*FAD2-2* (strain fad2MF2, Table 2) only restored the production of 18:2 (Fig. 4b). Introduction of Lu*FAD3-2* (strain fad2LF3, Table 2), had little effect on fatty acid profile (Fig. 4b). 18:3 was produced only when Ma*FAD2-2* and Lu*FAD3-2* were co-expressed (strain fad2ML, Table 2) (Fig. 4b). As expected, re-introduction of the endogenous *FAD2* gene into *fad2Δ* (strain fad2FAD2*,* Table 2) restored the biosynthesis of UFAs (Fig. 4c). Collectively, Fad2 is a bifunctional FAD with Δ12 and Δ15 activities.

### *FAD4* encodes a minor multi-functional desaturase with low regioselectivity

**Fig. 5:**
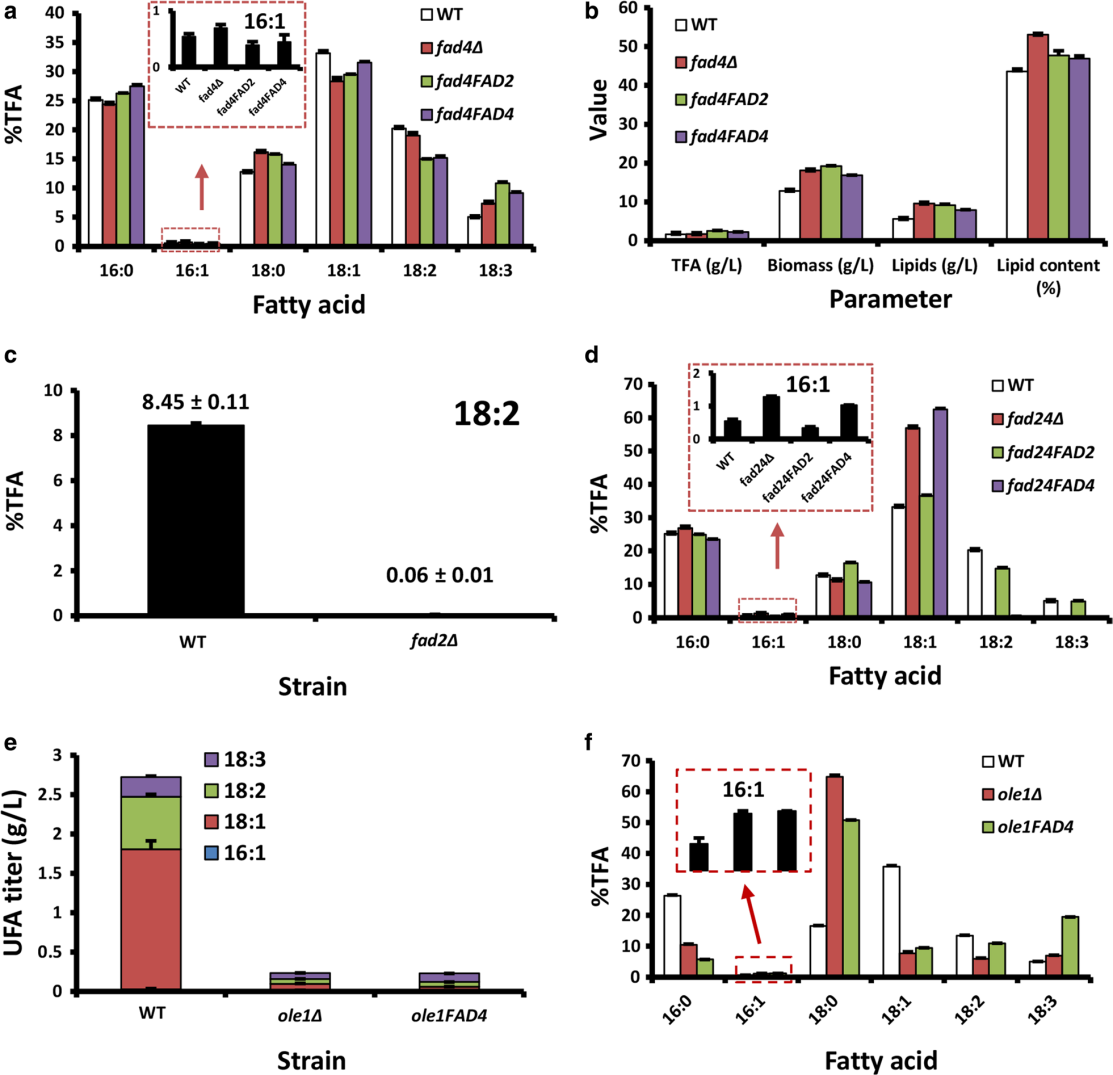
Identification and characterization of *FAD4*. **a** Fatty acid profiles of WT, fad4Δ, fad4FAD2 and fad4FAD4. **b** Biomass, lipid titers and lipid contents of WT, fad4Δ, fad4FAD2 and fad4FAD4. **c** 18:2 production (%TFA) in WT and fad2Δ strains. **d** Fatty acid profiles of WT, fad24Δ, fad24FAD2 and fad24FAD4. **e** Unsaturated fatty acid yields (titer, g/L) in WT, ole1Δ and ole1FAD4 strains. **f** Fatty acid profiles in WT, ole1Δ and ole1FAD4 strains. The inlet shows the profile of 16:1. %TFA represents weight percentage of total fatty acids. Error bars represent standard derivations of triplicates

The primary structure of Fad4 is more related to Δ4, Δ5, Δ6 and Δ8 FADs (Additional file 1: Fig. S3). However, there was no Δ6 (e.g., γ-linolenic acid, GLA) or Δ8 fatty acid detected in *R. toruloides* oil. Fad4 and Fad2 share 20.5% identity at amino acid level and 44.9% identity at cDNA level, suggesting that *FAD4* may be derived from gene duplication or horizontal gene transfer from *FAD2*-related gene. Due to the presence of a di-proline motif at the N-terminus (P^3^-P^4^), Fad4 might be destabilized by membrane fatty acid desaturation [54]. To investigate its function, *FAD4* null mutant (*fad4Δ*) was generated (Additional file 1: Fig. S5c). Deletion of *FAD4* resulted in a significant drop in 18:1 (14.7%, p < 0.001) and 18:2 (6.1%, p < 0.001), suggesting that Fad4 has Δ9 and Δ12 FAD activities (Fig. 5a). However, it is puzzling to see the rise of 18:3 in *fad4Δ* (Fig. 5a)*.* The increase of 18:0 level (Fig. 5a) might have resulted from pathway overflowing due to the downstream blockage.

To further investigate Fad4 function, *FAD2* and *FAD4* were overexpressed in *fad4Δ* (strain fad4FAD2 and fad4FAD4, respectively, Table 2) using the strong *GPD1* promoter. The increase of 18:3 level upon overexpression of either *FAD2* or *FAD4* strongly suggests both enzymes have the bifunctional ∆12/∆15 FAD activity (Fig. 5a). The drop of 18:2 level was likely the result of substrate consumption by the ∆15 FAD activity. Notably, the lack of *FAD4* significantly enhanced lipid content and cell mass production (Fig. 5b), suggesting its role in suppressing lipid biosynthesis and cell growth.

These results prompted us to re-examine the fatty acid profile of *fad2Δ*. Indeed, trace amount of 18:2 (0.06 ± 0.01% TFA) was found, accounting for 0.7% of wild-type strain (Fig. 5c). Thus, we created a double mutant for *FAD2 and FAD4* (*fad24Δ*, Table 2). As expected, 18:2 disappeared completely in *fad24Δ* (Fig. 5d). Overexpression of *FAD2* in *fad24Δ* (strain fad24FAD2, Table 2) largely restored the fatty acid profile. Significantly, 18:2 and 18:3 levels were increased at the expense of their precursor 18:1 (Fig. 5d), reinforcing our conclusion for the Δ12/Δ15 FAD activity of Fad2. In contrast, *FAD4* overexpression in *fad24Δ* background (strain fad24FAD4, Table 2) led to a slight increase of 18:1 (Fig. 5d), suggesting that Fad4 possessing a weak ∆9 FAD activity. To test this hypothesis, *FAD4* was overexpressed in *ole1Δ* (strain ole1FAD4, Table 2). Although this strain remained defective in growth (Fig. 5e and Additional file 1: Fig. S6), 18:1, 18:2 and 18:3 levels were all increased (Fig. 5f). Notably, the increase in 18:2 and 18:3 levels were not accompanied by a drop of 18:1, a phenomenon observed in Δ12/Δ15 FAD overexpression. This further implied that ole1FAD4 strain contained a weak Ole1-like activity. Collectively, our results suggest that Fad4 is an unusual enzyme with Δ9/Δ12/Δ15 trifunctional FAD activity. To the best of our knowledge, only one similar case has been reported to date, a Δ6 FAD with Δ9 and Δ12 FAD activity [55].

### Physiological roles of FAD

Being the major constituent, the number of double bonds in the fatty acids of phospholipids is critical for the physical property of cell membranes [56]. *OLE1* deletion led to almost complete halt of cell division (cell budding) (Fig. 3a), although cell morphology was little changed (Additional file 1: Fig. S7). It was puzzling that ∆9 MUFA (18:1), but not its saturated precursor (18:0) or further desaturation products (18:2 or 18:3), was critical for cell viability. Studies in animal cells show that OA is not simply a structural element of membranes; it plays complex signaling roles also [57].

**Fig. 6:**
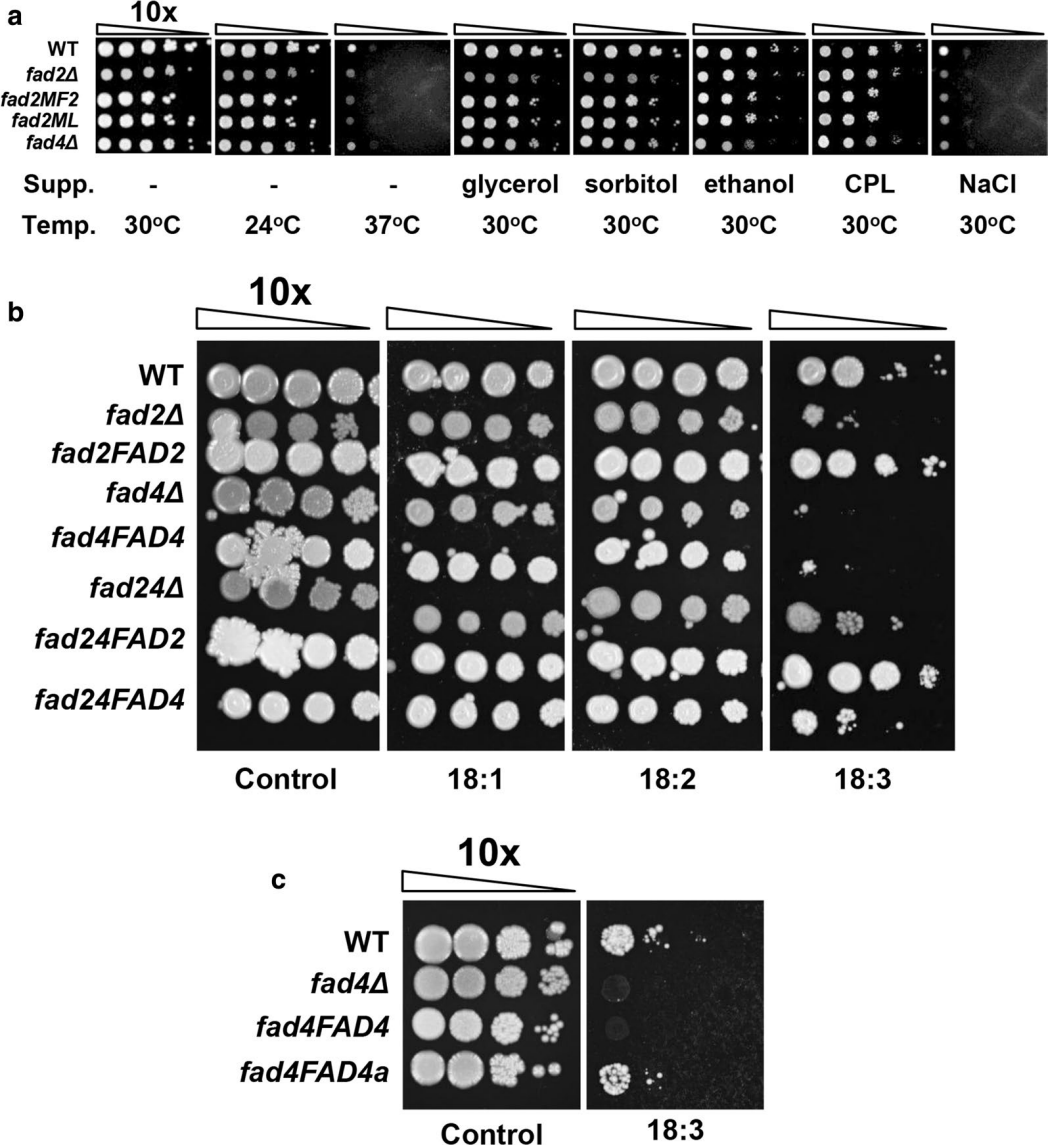
Stress responses. **a** Effects of various stress stimuli on cell growth. **b** Effects of unsaturated fatty acids on cell growth. **c** Effects of 18:3 on cell growth of *fad4Δ* and fad4FAD4a. Cell cultured at exponential phase were water-washed twice and spotted in tenfold serial dilutions on YPD agar plates supplemented with the indicated UFAs (0.1%, w/v) or stress-inducing chemical, and incubated at different temperatures. Cells cultured at 30 °C on YPD agar in the absence of any supplement was used as the control. fad4FAD4, fad4Δ harboring *FAD4* cDNA driven under *GPD1* promoter; fad4FAD4a, fad4Δ harboring whole *FAD4* allele; Supp., supplementation; Temp., temperature; CPL, β-caryophyllene. Concentrations used: glycerol, 2 M; sorbitol, 1 M; ethanol, 3% (w/v); CPL, 0.1% (w/v); NaCl, 0.8 M

*FAD2* deletion also led to slower cell growth under most conditions, which appeared to enhance the sensitivity to thermal stress (37 °C and 24 °C) and osmotic stress (glycerol or sorbitol) (Fig. 6a). As expected, the growth defect of *fad2Δ* was relieved by genetic complementation with a heterologous Δ12 FAD (strain fad2MF2) or Δ12 + Δ15 FAD (strain fad2ML, Fig. 6a).

Although Fad4 displayed weak activity in fatty acid desaturation, deletion of the gene significantly enhanced cell sensitivity to PUFA (18:3 in Fig. 6b). It is believed that ethanol alters plasma membrane transport [58] and increases membrane fluidity [59]. This is consistent with the previous observation that high unsaturation of fatty acids correlates with high cytotoxicity [60]. Surprisingly, overexpression of *FAD4* cDNA under the strong *GPD1* promoter only slightly relieved 18:3 sensitivity (Fig. 6b). On the other hand, re-introduction of the genomic *FAD4* allele to *fad4* mutant (*fad4FAD4a*) successfully complemented the growth defect (Fig. 6c), suggesting the significant role of introns in regulating *FAD4* expression. This is consistent with our earlier report on other genes in this host [26]. Taken together, Fad2 and Fad4 both play important roles in protecting cells from membrane stress. These findings open a new avenue to enhance fatty acids and terpenoid productivity in *R. toruloides*.

### *OLE2* encodes a weak regulator of Ole1

**Fig. 7:**
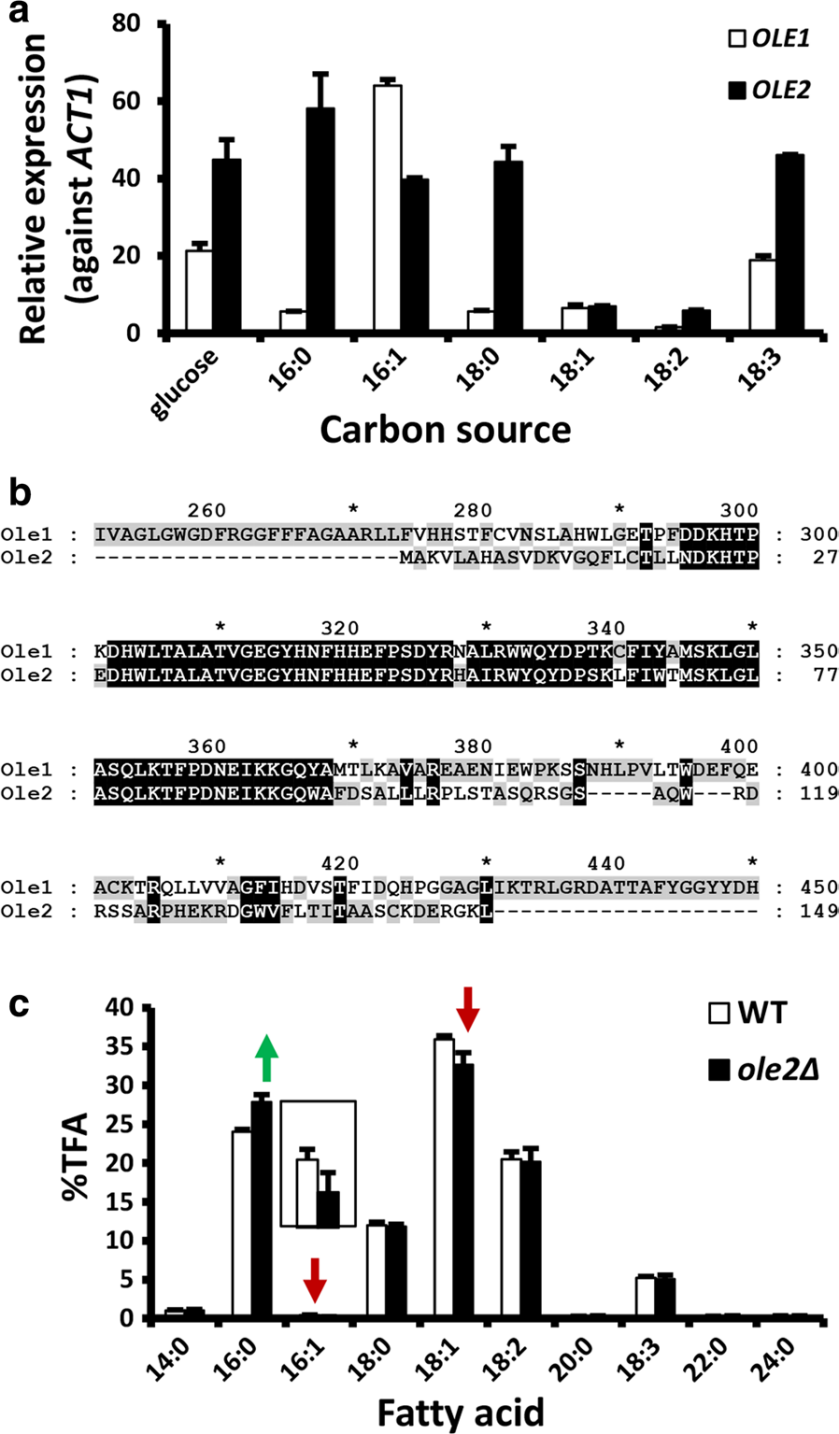
Identification and analyses of *OLE2*. **a** qRT-PCR analysis of *OLE1* and *OLE2* expression. Seed cultures were water-washed twice and inoculated in YNB broth (without carbon source) supplemented with different fatty acids (10 g/L) and Tergitol NP40 (1%, w/v), and cultured at 28 °C, 280 rpm for 8 h. Glucose (10 g/L) was used as the control. The values presented are the relative expression against *ACT1* mRNA (2-ΔCt method). Error bars represent the standard derivations of triplicates. **b** Sequence alignment of Ole1 (partial) and Ole2. **c** Fatty acid profiles of WT and *ole2Δ*. 16:1 was shown in the inlet plot

During the annotation of *OLE1*, another DNA fragment sharing high homology to *OLE1* was found in the genome. The 240-nt sequence shares 86% identity with *OLE1*. We tentatively named the gene *OLE2* (Table 1)*.* The conservation of this gene in different isolates, such as ATCC 10,788, NP11 and C3 strains [34, 61], suggest that it is functionally important. qRT-PCR analysis showed the sequence was abundantly transcribed and the transcripts level was regulated by fatty acids (Fig. 7a). In strain ATCC 10657, the sequence is located ~ 13 kb from *OLE1*. Notably, the predicted 149-aa Ole2 protein exhibits high identity to the region around the 3^rd^ histidine box of Ole1, a region believed to be crucial for regioselectivity of FADs (Fig. 7b). Targeted deletion of *OLE2* (Additional file 1: Fig. S5d) showed that 16:1 level dropped by 46.5% (*p* < 0.01) while 16:0 level increased by 16% (*p* < 0.05) (Fig. 7c). The levels of most other fatty acid species were not changed significantly. Deletion of *OLE2* did not appear to affect cell growth significantly. Thus, *OLE2* encodes a weak regulator of fatty acid desaturation, modulating the regioselectivity of Ole1. The effects of *OLE2* deletion were consistent with the overexpression of *OLE1* as both preferentially affected 16:1 level. Considering the strong role of 16:1 in inducing FAD gene transcription, it is possible that Ole2 can also regulate the transcription of *OLE1*, *FAD2* and *FAD4* indirectly.

### Metabolic engineering of fatty acids

**Fig. 8:**
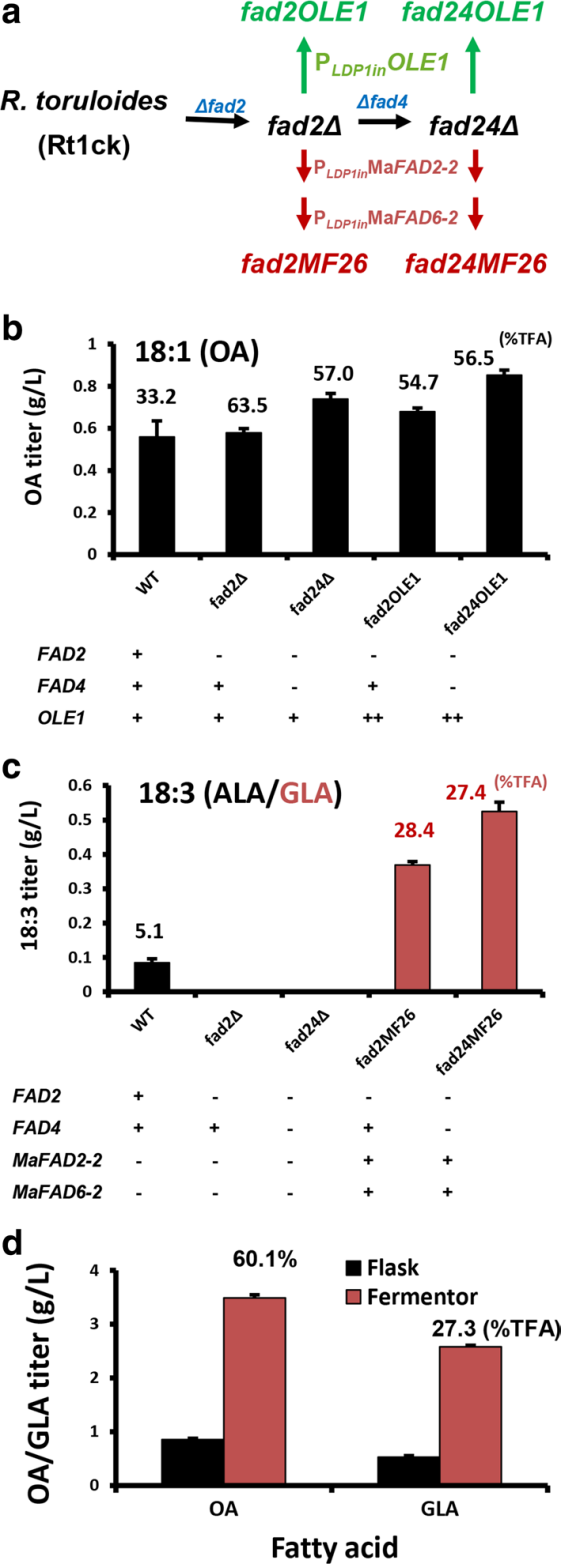
Metabolic engineering for oleic acid (OA) and γ-linolenic acid (GLA). **a** Metabolic engineering routes for production of OA and GLA. More information on the promoters and strains are shown in Table 2. **b** Production titer of OA of various strains. **c** Production titer of GLA of various strains. All strains were cultured in GJm3 medium for 5 days in shake flasks (30 °C and 250 rpm). The numbers shown on top the column show the weight percentage of total fatty acids (%TFA) of OA (in b) or GLA (in c). ALA is the α-linolenic acid produced in wild-type strain. Error bars represent standard derivations of triplicates. Symbols “ + ” and “−” represent the presence and absence of the gene, and “ +  + ” represents the presence of two copy of the gene. **d** Comparison of OA and GLA yields by flask and bioreactor fermentation

As a proof of concept, we demonstrated how the FAD genes could be exploited for high-level production of OA and a novel fatty acid, γ-linolenic acid (GLA) (Fig. 8a). High OA oil has many applications, including food, cosmetics, textiles, adhesives and biofuels [62, 63]. Deletion of *FAD2* led to little change in the volumetric productivity (titer) of OA, however, it significantly increased OA content, from 33.2 to 63.5% TFA (Fig. 8b). Further deletion of *FAD4* resulted in a 1.3-fold improvement in OA titer, although OA content was slightly decreased (Fig. 8b). To further improve OA production, the endogenous *OLE1* was overexpressed using the strong and lipogenic *LDP1in* promoter [26], resulted in a  strain with two copies of *OLE1* in the genome (mutant fad2OLE1 and fad24OLE1, Table 2 and Fig. 8a). This resulted in increased OA titer (15 ~ 17%, Fig. 8b). Surprisingly, OA content was reduced in *fad2Δ*, but not *fad24Δ* background (Fig. 8b). This difference may result from the interplay between cell growth mediated by the *FAD4* gene and fatty acid imbalance conferred by *OLE1* overexpression. Notably, increased expression of *OLE1* was reported to be toxic to the cells in *S. cerevisiae* [48].

*M. alpina* is a natural GLA producer, and its Δ12 and Δ6 FAD catalyze the final two steps of GLA biosynthesis [64]. Overexpression of *M. alpina* Ma*FAD2*-2 (synthetic Δ12 FAD) along with Ma*FAD6-2* (synthetic Δ6 FAD) (Fig. 8a) in *fad2Δ* and *fad24Δ* (mutant fad2MF26 and fad2MF26, Table 2) successfully turned *R. toruloides* into a GLA producer, resulting in a titer of 0.37 g/L (28.4%TFA) and 0.53 g/L (27.4%TFA), respectively (Fig. 8c). A preliminary 2-L-scale fed-batch fermentation showed that the maximal OA and GLA titer reached 3.5 and 2.6 g/L, representing 60.1% and 27.3% TFA, respectively (Fig. 8d).

Recently, an engineered *Y. lipolytica* strain has been reported to produce GLA to 4.6% of total fatty acids. The low yield could have resulted from the toxicity of GLA as lowering the culturing temperature increased GLA yield about 61% [65]. Notably, our GLA content has far exceeded the dominant commercial product, evening primrose oil [12]. We expect further improvement in OA and GLA yields when PUFA degradation and fatty acid selectivity in TAG synthesis can be manipulated. Thus, *R. toruloides* can be a strong platform for PUFA metabolic engineering and production.

## Conclusion

*R. toruloides* genome encodes a single-copy highly conserved ∆9 FAD, which is essential for cell viability and biosynthesis of MUFAs and PUFAs. The mutant *ole1Δ* provided a rare genetic insight into the role of Δ9 FAD on cell growth, fatty acid desaturation and lipid accumulation. As a yeast that is phylogenetically distant to the popular yeast hosts, such as *S. cerevisiae* and *Y. lipolytica*, it was not surprising to see *R. toruloides* has evolved significantly in the control of fatty acid biosynthesis and FAD gene expression, which were exemplified by the regulation of gene transcription via the GC-rich ORE1 motif, preferential induction by palmitoleic acid and involvement of two similar multi-functional FADs for PUFA biosynthesis. Fad4 is particularly interesting, not only for its relaxed regioselectivity of fatty acid desaturation, but also its roles in stress tolerance and maintaining healthy biomass and lipid production. Our data should illuminate PUFA engineering beyond this host.

## Materials and methods

### Strains, media, and culture conditions

Strains used are listed in Table 2. *R. toruloides* strain ATCC 10657 was obtained from ATCC (USA), and strain C3 was isolated from a fish sample in Singapore. Both are haploid (mating type *A1*) and share high genome sequence homology to *R. toruloides* ATCC 204091 (GenBank No. AEVR02000000) [34, 66]. *R. toruloides* strain Δku70e, a *KU70* null mutant with high frequency of homologous recombination [47], is referred as the wild-type strain in this study. Yeast strains were maintained at 28–30 °C in YPD broth (1% yeast extract, 2% peptone, 2% glucose, w/v) or on potato-dextrose agar (PDA, Sigma-Aldrich, USA). YPDtO is YPD broth supplemented with 0.1% (w/v) oleic acid and 0.5% (w/v) Tergitol NP40) and was used for propagation of *ole1Δ*.

GJm3 is a lipid accumulation medium modified from the previous report [15]. It contains (per liter) 70 g glucose, 2.5 g yeast extract, 0.4 g KH_2_PO_4_, 1.5 g MgSO_4_·7H_2_O, 40 mg CaCl_2_·2H_2_O, 5.5 mg FeSO_4_·7H_2_O, 5.2 mg citric acid·H_2_O, 1 mg ZnSO_4_·7H_2_O, 0.76 mg MnSO_4_·H_2_O and pH was adjusted to 6.0 with sulfuric acid.

Yeast nitrogen base (without amino acid or ammonium sulfate) containing glucose (20 g/L) was used as nitrogen starvation medium (YNB-N^−^) while nitrogen rich medium (YNB-N^+^) was YNB-N^−^ supplemented with 5 g/L ammonium sulfate. Cells were cultured until exponential phase; washed twice with water; inoculated to either YNB-N^−^ or YNB-N^+^ and cultured at 28 °C with 280 rpm agitation. In fatty acid supplementation experiments, *R. toruloides* cells were cultured in YNB broth containing 5 g/L ammonium sulfate, a fatty acid of interest or glucose (10 g/L) and Tergitol™ NP40 (1%, w/v).

### DNA constructs

DNA constructs used are listed in Table 2. Oligonucleotide sequences are listed in Additional file 1: Table S2. DNA constructs were verified by restriction mapping and DNA sequencing using the BigDye method (ABI). Details for DNA vector construction are shown in Additional file 1: Fig. S8.

### Extraction of genomic DNA and total RNA

Genomic DNA and total RNA extraction were performed as reported previously [25]. Nucleic acids were quantified using NanoDrop® ND-1000 Spectrophotometer (NanoDrop Technologies, Wilmington, USA) and the quality was checked by agarose gel electrophoresis.

### Rapid amplification of cDNA ends (RACE)

5′ RACE and 3′ RACE were done using BD SMARTer™ RACE cDNA Amplification Kit (BD CLONTECH Laboratories, Palo Alto, CA, USA) according to the manufacturer’s instructions. Oligo pair OLE1U1/OLE1L1, FAD2U1/FAD2L1 and FAD4U1/FAD4L1 (Additional file 1: Table S2) was used as the specific primer for 5′ and 3′ RACE of *OLE1*, *FAD2* and *FAD4*, respectively.

### Gene annotation and phylogenetic analysis

As the genome sequences of *R. toruloides* strain ATCC 10657, IFFO 0880 and ATCC 204091 [34, 66] are highly similar, the annotated genome database of *R. glutinis* ATCC 204091 was used as the reference. FAD genes were identified using tBLASTn at NCBI against the reference genome database using protein sequences of various types of well-characterized FADs as the queries. Full-length cDNA sequences were obtained by RT-PCR after the 5′ and 3′ ends were determined by RACEs. The FAD orthologs of other *Pucciniomycotina* species were predicted by BLASTp program. Sequence alignment and phylogenetic analysis were performed with the MEGA 6 program (www.megasoftware.net) using the Neighbor-Joining algorithm [67]. The membrane configurations of the proteins were predicted at the transmembrane prediction server TMHMM-2.0 (http://www.cbs.dtu.dk/services/TMHMM/). The consensus sequences were analyzed through the MEME suite server (http://meme-suite.org/) [46].

### Genetic manipulation

*Agrobacterium tumefaciens*-mediated transformation (ATMT), targeted gene deletion and fungal colony PCR were performed as described previously [25, 47]. Oleic acid was supplemented to the media for co-culture and selection in order to obtain *OLE1* the deletion mutant while LA was supplemented to culture media to facilitate the generation of *FAD2* and *FAD4* mutants.

Gene expression cassettes were usually site-specifically integrated to the *CAR2* locus (encoding phytoene synthase/lycopene cyclase) to eliminate positional effects. Knock-in mutants were selected based on the albino phenotype [26, 47, 68]. At least 3 biological replicates were used in the assays.

### Southern blot analysis

Genomic DNA (5 µg) was digested with a restriction enzyme and separated by electrophoresis in a 0.8% agarose gel. Southern blotting was performed as described using digoxigenin-labeled DNA as the probes [69]. The restriction enzymes and DNA probes used are shown in Additional file 1: Fig. S5a-d.

### Analyses of gene expression

Total RNA preparation, cDNA synthesis, real-time PCR analysis and luciferase gene reporter assay were performed as reported previously [26]. Briefly, *R. toruloides* strains harboring different reporter cassettes were cultured in YPD broth until exponential phase. Cells were cultured for 8 h in fresh YPD broth, which may be supplemented with a fatty acid of interest at 0.1% (w/v).

### Quantification methods

Quantification of cell biomass (dry cell weight), residual glucose and lipids were performed as previously reported [70]. Fatty acid profile was determined by gas chromatography–mass spectrometry (GCMS) after esterified to fatty acid methyl esters (FAMEs) as described previously [70]. The specific fatty acids were quantified by normalization against the internal standard (ISTD, 15:0) and the corresponding response factor against ISTD as calculated through a pre-run of the standard FAME mixture (Supelco 37 Component FAME Mix, Sigma, USA).

### GenBank accession numbers

Based on the codon preference of highly expressed genes in *R. toruloides*, Ma*FAD2-2*, Ma*FAD6-2*, Lu*FAD3-2* were synthesized by GenScript (USA) according to the protein sequence of *M. alpina* FAD2 (ADE06660), *M. alpina* FAD6 (AAL73949), *L. usitatissimum* omega-3 desaturase (AFN53677), respectively. The nucleotide sequences have been deposited to GenBank under the accession number MF152712 through MF152717.

## Supplementary Information

**Additional file 1.** Additional tables and figures.

## Data Availability

The authors declare that all data supporting the findings of this study are available within the paper and its supplementary information files or from the corresponding author on request.

## Abbreviations

ATCC: American Type Culture Collection
CDS: Coding sequence
ER: Endoplasmic reticulum
16:1/POA: Palmitoleic acid (16:1Δ^9^)
18:1/OA: Oleic acid (18:1Δ^9^)
18:2/LA: Linoleic acid (18:2Δ^9,12^)
18:3/ALA: α-Linolenic acid (18:3Δ^9,12,15^)
GLA: γ-Linolenic acid (18:3Δ^6,9,12^)
FAD: Fatty acid desaturase
Ole1: Δ9 Stearoyl-CoA desaturase
Fad2: Δ12/Δ15 Bifunctional fatty acid desaturase
Fad4: Δ9/Δ12/Δ15 Trifunctional fatty acid desaturase
*OLE2*: *OLE1*-Related gene
RACE: Rapid amplification of cDNA ends
TFA: Total fatty acids
UTR: Untranslated region
