# Supplementary material for: Understanding and exploiting the fatty acid desaturation system in Rhodotorula toruloides
Source: Biotechnol Biofuels. 2021 Mar 19;14:73. doi: 10.1186/s13068-021-01924-y (PMC7977280; doi:10.1186/s13068-021-01924-y)
Supplement: Supplementary file 1 — Additional file 1. Additional tables and figures. [file 13068_2021_1924_MOESM1_ESM.docx]

# Supplementary information

# Understanding and exploiting the fatty acid desaturation system in *Rhodotorula toruloides*

Yanbin Liu**^1^**, Chong Mei John Koh**^1^**, Sihui Amy Yap**^1^**, Lin Cai**^1^** and Lianghui Ji**^1,2,*^**

**^1^**Temasek Life Sciences Laboratory, 1 Research Link, National University of Singapore, Singapore 117604

**^2^**School of Biological Sciences, Nanyang Technological University, Singapore, 60 Nanyang Drive, 637551, Singapore

^*^Corresponding authors

Phone: +65-68727483

Fax: +65-68727007

Email: jilh@tll.org.sg (LJ)


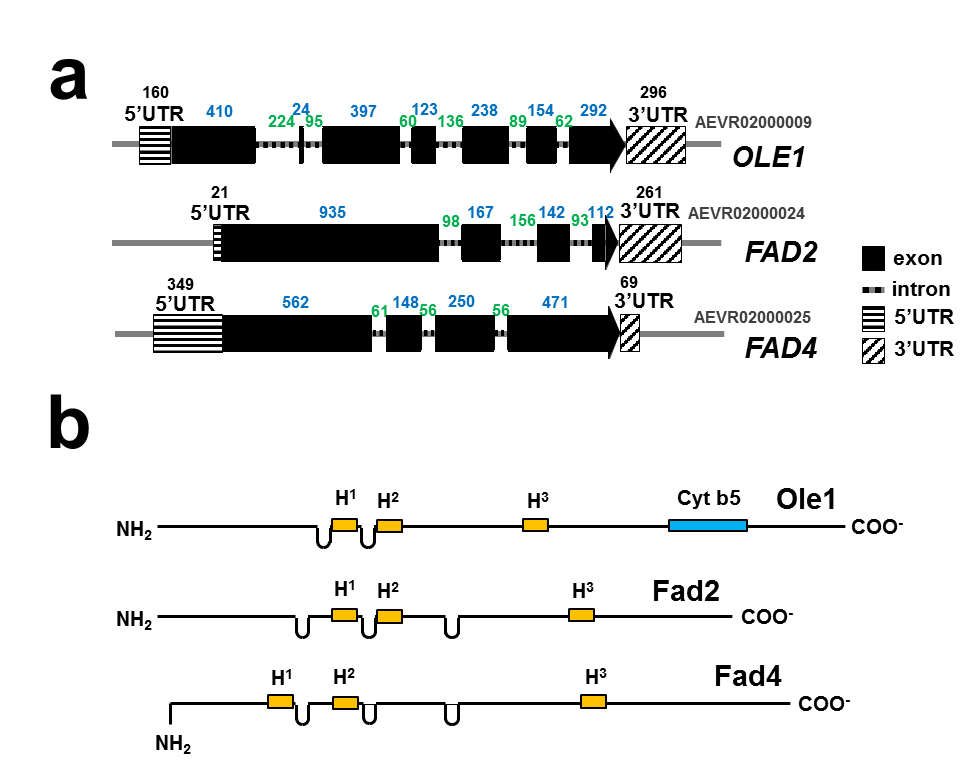


## Additional file 1: Fig. S1. Fig. S1. Gene structure and protein sequence alignment of FADs in *R. toruloides*. (a) Schematic diagram of FAD gene structures in *R. toruloides*. The length of 5’/3’ untranslated regions (5’UTR/3’UTR), exons and introns were labeled in black, blue and green font by the unit of nucleotides. (b) Structure and topology of *R. toruloides* FADs. Protein sequences of Ole1, Fad2 and Fad4 were predicted at TMpredit server (Expasy, https://embnet.vital-it.ch/software/TMPRED_form.html). The histidine-boxes were boxed in yellow color, and the cytochrome b5-like domain was boxed in blue color. The N- and C-termini were labelled with NH_2_ and COO^-^, respectively.


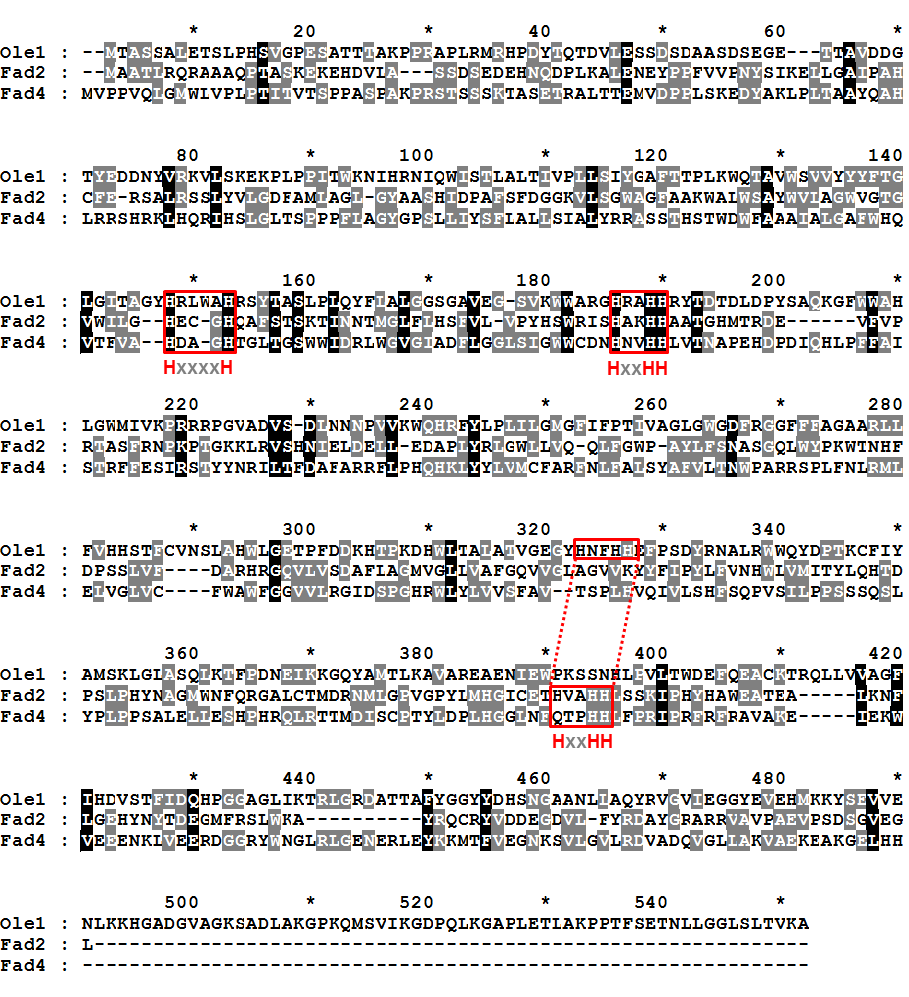


## Additional file 1: Fig. S2. Protein sequence alignment of FADs in *R. toruloides*. The three representative histidine motifs were boxed in red.


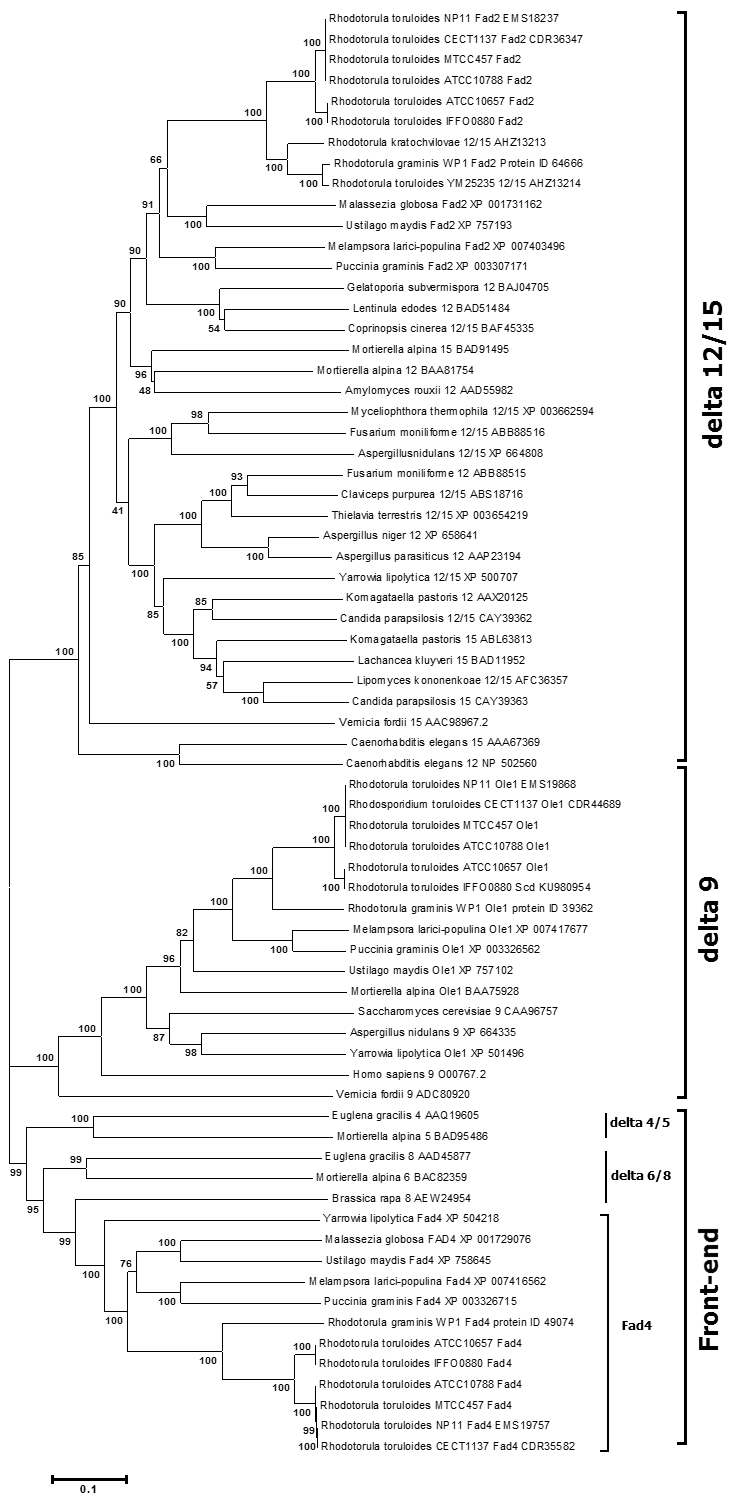


## Additional file 1: Fig. S3. Phylogenetic analysis of fatty acid desaturases. Phylogenetic tree was constructed with MEGA version 6 using Neighbor-Joining algorithm. Enzyme name was represented as “Taxonomy name (strain name)” + FAD name or function position” + “GenBank accession number or protein ID in the genome sequence database”.

## Additional file 1: Table S1. Comparison of FADs from different mating types

| **Gene** | **CDS identity (%)** | **Protein identity (%)** | **Protein similarity (%)** |
| --- | --- | --- | --- |
| *OLE1* | 88.9 | 97.2 | 98.9 |
| *FAD2* | 88.1 | 96.9 | 98.7 |
| *FAD4* | 87.1 | 94.3 | 96.4 |

Note: FAD sequence of mating type α strains (ATCC 10657 and IFFO0880) was compared against mating type A strains (ATCC 10788, MTCC 457, CECT 1137 and NP11).


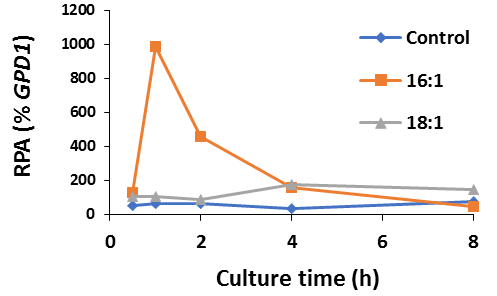


## Additional file 1: Fig. S4. Effect of OA and POA on *OLE1* promoter activity. A promoter luciferase gene reporter strain was created using 843 bp *OLE1* promoter and 35S terminator, which was knocked in at the *CAR2* locus and cultured in YPD medium (Control), or YPD medium supplemented with POA (16:1) or OA (18:1). Cells were sampled at the culture time indicated. The relative promoter activity (RPA) shows the reading standardized against that of *176 bp GPD1* promoter reporter strain.

## Additional file 1: DNA sequences of *OLE1*, *FAD2* and *FAD4*. The number in the bracket indicates the sequence length (translational start codons are underlined).

>*OLE1* (843 nt)

tcctcgaccccatgcgaaggcgagctctgctcgtcagctggccaagctggccagcagacgagcgttggggtggcggaacgccaacggcatggagtaaagcagccgtgaggatgacggaggagctcgggcgaggtgatggggattctagcaggaacagcagagcggcgaggaggagaggaaccggaagcacagtctcgtggccgcttgttgcagatcccagtgtcgctagagtgctcgtcgtcatcagagcgagtgaacaaagcgatgccctgaagaacgatgagcgaatgagtcgaagcggcgtctaccggtgaactcggggtgtggcaaatgagcgagacgaggagtgcccgccagagttgccacgtcgaccccacgtcggaatcgacgttgatagagtgaacgaagccattgcagaccccagaaggtggccatgttgtggaagcgagggcaggagcgaggggagaaggcgaggaggaggaggggctggggaagcccgtccgggaatggcgcagctgggtgccggggatgtgcgcgagtggcggaggagtcgagcgtgagagttctggaacacggggcgcgcacaagggtcgagggccgtgacgagttcgccgggcggtggtcgggctgagggcgagcgcgcgttggggacgacgacgcccgacgccctcgctcttcgtcctcaccgcttcccggagaactttgctgtactctgcttctcccttcacactctcacacccactcacacacccttccatccacacacaagctatccgcacacctctcacacccgaccccagctcgccccatcctcttcgcacccggctcatcgccacacacgcaatg

>*FAD2* (339 nt)

cttgtcccggtcagcaacggaatgtctcgcctgtgcttagtgccgtgcccaggccgccgtttgattcgccggttcgcggttcgcgcccatcgcgcgagggagagcttcgaccgctcgttcgcctttctccttcacccgctcactccgccccgcttacccttctctctcccttagactcgtgtgagtgtgatagcactgttatcaatcagcgcacgacactgacagcacgcggaccacgtcgaacagcgcagacccctgcacgtttcccctccgcacaacccccaacccccctccctccccgctcgaccggccccctccgactgcacagcgacaccagcaatg

>*FAD4* (818 nt)

gctggatggaagaacctcgctaatgctcggttcgctcacagtgccgccgccctcgagcagggcaaggccgccaagaaggagaaggagtcgaagaaggtcaagaacccggccaacaaggtcccccagggcgcaaaggtctcgcgcgctcaggctggaaagggcgccgttcgctccggccgttaatttccctggagacgcggtggtggaccttgcggtgtgcaacggttgttgaaactctttccttgggattgacgagagaagggagcggcttcgctgggttcggcgttgcttcgaggctgttttgctaacggtccttgtcaatgcttgcaagccccgctaggccacaacgtcgccgtcgtcgagagcagcacacgttgcgtctcgtcctctgctcgtctcgctccgtccgctcaccgctacttgcttccacggtctcgccgggctttctgagggcttgcacagtgccacagacgcagctgggcaccatggcagacgcgaccgccgccccgccgccctctcctcactccccatctcccaccgccgcaccctcctcctcgcgcctctcctcccgcctaacctgcgccggcgtgcccctcccatcgaatttctccaccctctcgaccctctcgcgccccgaaatcgcctcccgcataacccgcggcgagttactcgtcctccaccctcctctcgtctatcgcatccctcaatcctggcttcgcctccacccgggcggacaacactcgatcctgcactatgtaggccgggacgcgagctgcgagattgaggggtaccattcgggccggactgtgagtgagaggatg

>*OLE1* terminator (3’ UTR is shown in bold font)

**tggcccgtcaccgccatcggttcaagggagagtcgtcgaccgactcttccgttcgtcgcttcaggttcatccgtcttcgcatgcttagagaccctcttcctcgttgttttgtgcatcagttgcccgcagtcgtcccttaacgagcccactactaccctttccatgcccagtattcttgcccccgcccccttcgttctttgatacgtccaaccacctcgctctttcgctgtagctctacttcgttctctcccttgc**gggttgcccgcttactccgcatggaaacttgaccagtgtgcacaatactcttgcgactcgtccagcgctgcgag


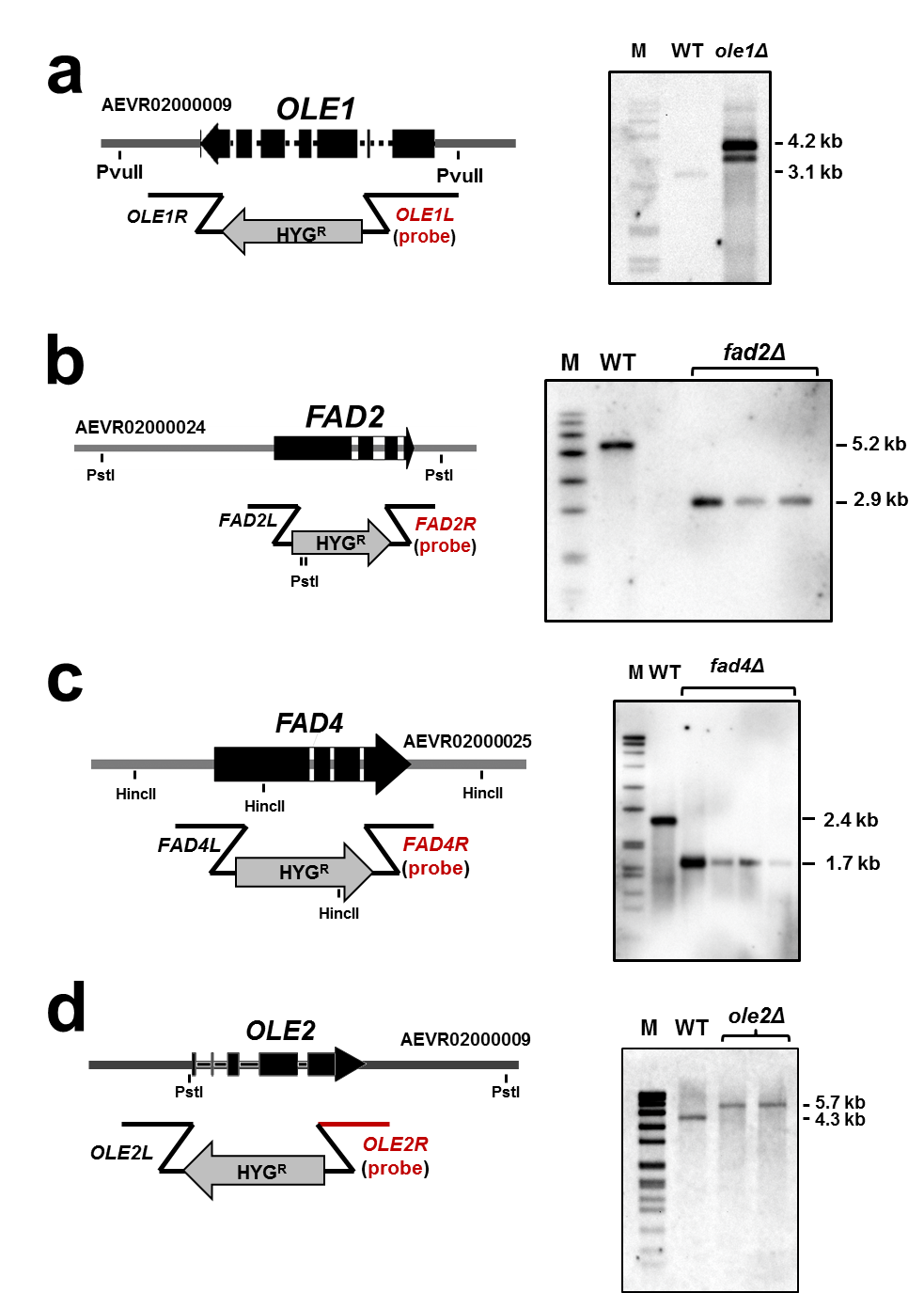


## Additional file 1: Fig. S5. Scheme of gene deletion strategies and Southern blotting. (a) *ole1Δ*. (b) *fad2Δ*. (c) *fad4Δ*. (d) *ole2Δ*. For Southern blotting, Genomic DNA was digested with indicated restriction enzymes and hybridized with the DIG-probe of DNA fragment in red font. M: DNA Molecular Weight Marker VII, DIG-labeled (Roche Diagnostics, Mannheim, Germany).

## Additional file 1: Fig. S6. Cell growth phenotypes of *ole1Δ* and *ole1FAD4*. Strains were streaked on PDA media in the presence (+) or absence (-) of oleic acid (0.1%, w/v) and incubated for 3 days at 28^o^C.


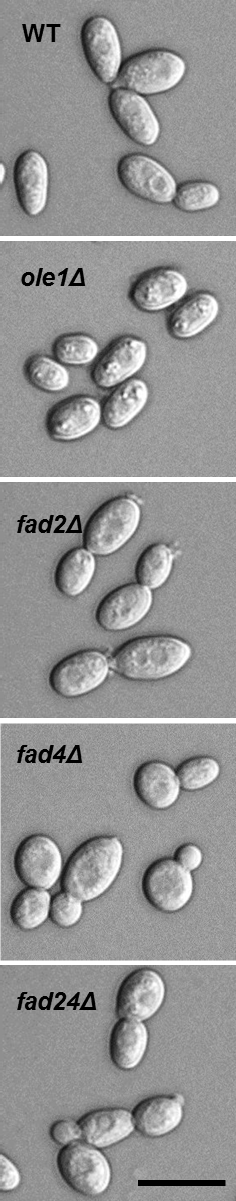


## Additional file 1: Fig. S7. Cell morphology of FAD mutants. All strains except *ole1Δ* were cultured in YPD medium at 30ºC till exponential phase and imaged under differential interference contrast (DIC) microscopy. *ole1Δ* was cultured in YPD medium supplemented with oleic acid till exponential phase. Cells were harvested by centrifugation, water-washed twice and cultured in fresh YPD medium for 12 h before image was taken. Scale bars represent 10 μm.

## Additional file 1: Table S2. Oligonucleotides used

| **Name** | **Sequence*** | **Application** | **Restriction enzyme** |
| --- | --- | --- | --- |
| ***RACE specific oligos*** | | | |
| OLE1U1 | GACCGCCGTCTGCCACTTCAG | *OLE1* |  |
| OLE1L1 | CTCGCCAGCTCCTCGTTGTCG |  |  |
| FAD2U1 | GCGTCCGAGACGAGGACCTGAC | *FAD2* |  |
| FAD2L1 | CCAACCACTTCGACCCGTCGTC |  |  |
| FAD4U1 | GCCCTCCCCTCCCTCACTCTCA | *FAD4* |  |
| FAD4L1 | CTTCTGGGCGTGGTTTGGAGGA |  |  |
| ***Deletion of OLE1*** | | | |
| DS9L-Sf | GCGAGGGATGGCAGTAAGACG | *OLE1L* | SacI |
| DS9L-Br | AAA*ggatcc*AACTTGCTCGCCCAGTACC |  | BamHI |
| DS9R-Hf | TTT*aagctt*CACGTACAGCCTGTGGTAGCC | *OLE1R* | HindIII |
| DS9R-Str | TTT*aggcct*GGAGGAGTCGAGCGTGAGAGT |  | StuI |
| DS9f | CTGCTCGCCTCCTCTTTGTCC | Colony PCR |  |
| DS9r | ACAGGCAAGTGGTTCGACGAC |  |  |
| ***Deletion of FAD2*** | | | |
| DS12L-Sf2 | AAA*gagctc*GGTGACTGCATGCTCCGTTAC | *FAD2L* | SacI |
| DS12L-Br2 | AAA*ggatcc*TGATGGAGTAGTTGGGCACGA |  | BamHI |
| DS12R-Hf | TTT*aagctt*CCTCCTCCTTGATCTTTCGCCG | *FAD2R* | HindIII |
| DS12R-Str | TTT*aggcct*GACCTTTGCGTCCTCCCTTCA |  | StuI |
| DS12-2f | CTGTACGTCCTCGGCGACTTT | Colony PCR |  |
| DS12-2r | GGTCCACTTGGGGTACCAGAG |  |  |
| ***Deletion of FAD4*** | | | |
| DS6L-Sf | AAA*gagctc*CTCACTCCCCATCTCCCAC | *FAD4L* | SacI |
| DS6L-Br | AAA*ggatcc*AGAACGCACGATGATGCACGT |  | BamHI |
| DS6R-Pf | TTT*ttaattaa*TCGATCCTCCCTCCTTCTTCCT | *FAD4R* | PacI |
| DS6R-Str | TTT*aggcct*GGCAATATCGTCTCCTGCACCT |  | StuI |
| DS6f | TGATAGGCTGTGGGGAGTCGG | Colony PCR |  |
| DS6r | GACTCGTCACCGCAAAGCTCA |  |  |
| ***Promoter analysis*** | | | |
| Rt864Sf | TTT*actagt*CGCAGGAAGCCGTTACAAGC | Promoter *GPD1-176* | SpeI |
| Rt897Sf | TTT*actagt*TGTTGCAGATCCCAGCGCAGGAAGCCGTTACAAGC | Promoter *ORE1-GPD1-176* | SpeI |
| Rt898Sf | TTT*actagt*CATTGCAGACCCCAGCGCAGGAAGCCGTTACAAGC | Promoter *ORE2-GPD1-176* | SpeI |
| Rt899Sf | TTT*actagt*TGTTGCAGACCCCAGCGCAGGAAGCCGTTACAAGC | Promoter *ORE1m-GPD1-176* | SpeI |
| Rt012N | TTT*ccatgg*TGAGTGATCTGGTGTTGTTC | *GPD1* related promoter | EcoRV |
| Rt848Sf | TTT*actagt*GCTTGTTGCAGATCCCAGTG | Promoter *OLE1-641* | SpeI |
| Rt434Nr | TTT*ccatgg*CGTGTGTGGCGATGAGCC |  | EcoRV |
| Rt862Evf | TTT*gatatc*TGGCCCGTCACCGCCATC | Terminator *OLE1* | EcoRV |
| Rt863Pmr | TTT*gtttaaac*TCGCAGCGCTGGACGAGTC |  | PmeI |
| ***Gene overexpression*** | | | |
| Rt227Nf | TTT*ccatgg*CTGCCTCGTCGGCACTCGAG | *OLE1* | NcoI |
| Rt228Evr | TTT*gatatc*CATTACGCCTTGACCGTCAG |  | EcoRV |
| Rt340Nf | TTT*ccatgg*TACCGCCTGTGCAGTTG | *FAD4* | NcoI |
| Rt284Pmr | TTT*gtttaaac*GACCAGCGCGCGTCTAGTG |  | PmeI |
| ***qPCR analysis*** | | | |
| qOLE1f | ACCTCGGCTGGATGATTGTC | *OLE1* |  |
| qOLE1r | AGCGACGATGGTAGGGAAGA |  |  |
| qDES12f | TCACCTACCTCCAGCACACG | *FAD2* |  |
| qDES12r | AGATGCCGTGCATCAGGTAA |  |  |
| qDES6f | CGGACTTTTTGGGAGGACTG | *FAD4* |  |
| qDES6r | GGGTCGAGATGGCAAAGAAC |  |  |
| qACT1f | GAGGGCGACGTGATCCTGTTC | reference actin gene |  |
| qACT1r | GTAGACGACCGAAGCACCACC |  |  |

* Sequence in *italics* and lowercase denotes the restriction enzyme recognition site.


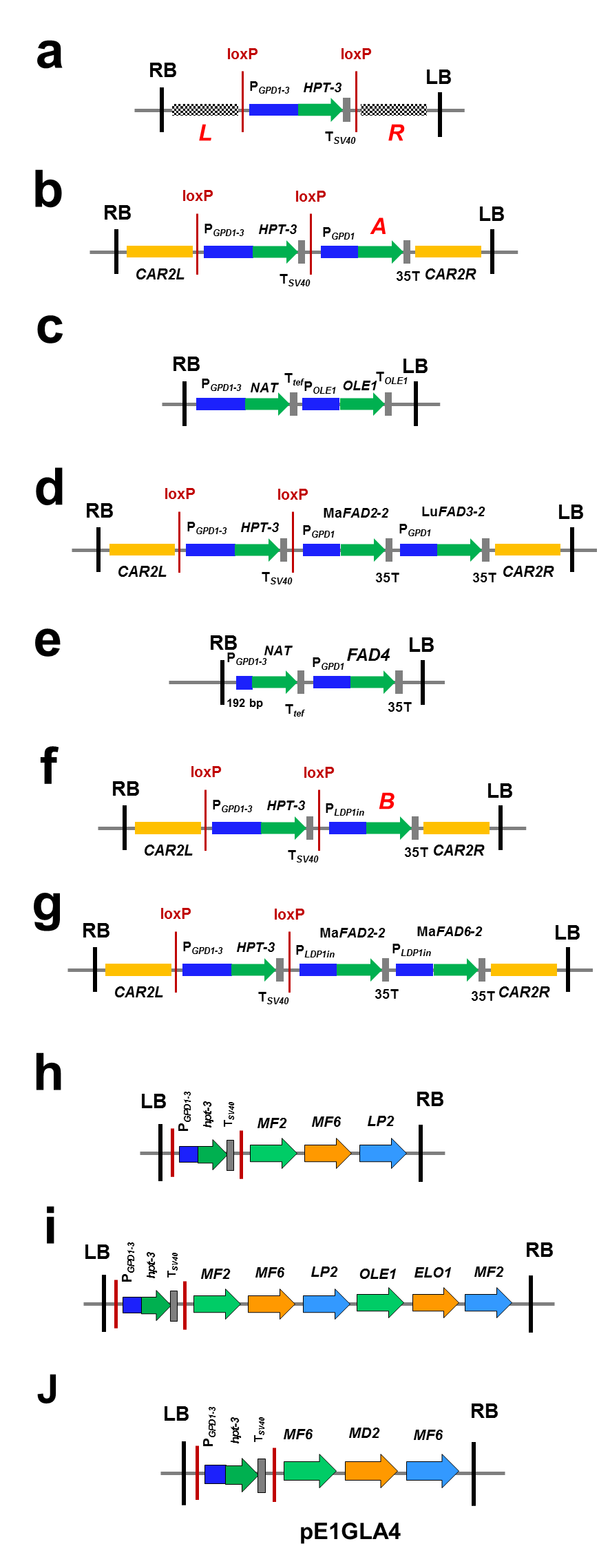


## Additional file 1: Fig. S8. Diagrams of T-DNA constructs. (a) Gene deletion plasmids pKOOLE1, pKOFAD2 and pKOFAD4, where the homology arms, *L* or *R,* represents OLE1L/OLE1R, FAD2L/FAD2R and FAD4L/FAD4R, respectively. (b) Gene expression plasmids pKC2OLE1, pKC2FAD4, pKC2MF2 and pKC2LF3, where *A* represents *OLE1*, *FAD4*, Ma*FAD2-2* and Lu*FAD3-2*, respectively. (c) Gene expression plasmid pKC2ML. (d) Plasmid pNEG1OLE1. (e) Gene expression plasmid pNEG1FAD4. (f) Gene expression plasmids pKP4OLE1, pKP4MF2 and pKP4MF6, where *B* represents *OLE1*, Ma*FAD2-2* (MF2) and Ma*FAD6-2* (MF6), respectively. (g) γ-linolenic acid (GLA) engineering plasmid pKP4MF26.

Gene knockout plasmids pKOOLE1, pKOFAD2 and pKOFAD4 were created by multi-fragment ligation of the NcoI-BamHI double-digested vector pEX2 [1] with SacI-BamHI double-digested 5’ homology arm of the target gene, BamHI-HindIII double-digested hygromycin resistance gene cassette (*Hyg^R^*, Table 1) and HindIII-StuI double-digested 3’ homology arm of the target gene.

DNA fragments used for luciferase gene reporter assay were cloned by PCR using genomic DNA of *R. toruloides* as the template. Promoter *GPD1-176*, *ORE1-GPD1-176*, *ORE1-GPD1-176* and *ORE1m-GPD1-176* was amplified using oligo pair Rt864Sf/Rt012N, Rt897Sf/Rt012N, Rt898Sf/Rt012N and Rt899Sf/Rt012N, respectively. The resulted DNA products were double digested with SpeI and NcoI, and inserted to pKCL2 at the same sites to create pKCL25, pKCL254, pKCL255 and pKCL256, respectively. Similarly, *OLE1* (641 bp) promoter was amplified using oligos Rt848Sf and Rt434Nr, and the PCR products was double digested with SpeI and NcoI, and inserted to pKCL2 at the same sites to create pKCLF66. Terminator of *OLE1* was amplified using oligos Rt862Evf and Rt863Pmr and the resulted PCR products was double digested with EcoRV and PmeI, and inserted to same sites of pKCLF66 to create pKCLF661.

To overexpress genes under the control of *GPD1* promoter [2] and the *Cauliflower mosaic virus* (CaMV) 35S gene terminator, the cDNA of *OLE1* and *FAD4* was amplified by reverse transcription PCR (RT-PCR) using oligo pair Rt227Nf/Rt228Evr and Rt340Nf/Rt284Pmr, respectively. The resultant PCR products was double-digested with NcoI/EcoRV and NcoI/PmeI, and inserted into the same sites of pKC2 [3] to create plasmid pKC2OLE1 and pKC2FAD4, respectively. Similarly, synthetic DNA Ma*FAD2-2* and Lu*FAD3-2* (Table 1) was linearized with NcoI-EcoRV and BspHI-EcoRV, respectively and inserted to pKC2 at the NcoI-EcoRV sites to create pKC2MF2 and pKC2LF3, respectively. To co-express Ma*FAD2-2* and Lu*FAD3-2*, the P*_GPD1_*-Ma*FAD2-2*-T*_35S_* cassette was released from pKC2MF2 using SpeI (blunt-ended) and PmeI and inserted at the PmeI site of pKC2LF3 to create pKC2ML. To complement *ole1Δ* mutant, plasmid pNEG1OLE1 was created by inserting wild-type *OLE1* allele (-641~+2733 from translational start site) to the SpeI (blunt-end) and SspI sites of pNEG1, and plasimd pNEG1FAD4 was created by inserting FAD4 cDNA to pNEG1 at NcoI and PmeI sites. To complement *fad4Δ* mutant, plasmid pKC2FAD4a was created by inserting wild-type *FAD4* allele (-884~+2128 from translational start site) to the SpeI and SspI sites of pKC2.

To overexpress heterologous FAD genes under the regulation of *LDP1in* promoter, pKP4OLE1, pKP4MF2 and pKP4MF6 were created. *OLE1*, Ma*FAD2-2* and Ma*FAD6-2* was inserted to the NcoI and EcoRV site of pKCLP4 [4], respectively. For production of GLA, pKP4MF2 was double-digested with SpeI (blunt-ended) and PmeI, and the resultant P*_LDP1in_*-Ma*FAD2-2*-T*_35S_* cassette was inserted to the PmeI site of pKP4MF6 to yield pKP4MF26, which contains both Ma*FAD2-2* and Ma*FAD6-2* gene expression cassettes.

**References**

1. Liu Y, Koh CM, Sun L, Ji L: **Tartronate semialdehyde reductase defines a novel rate-limiting step in assimilation and bioconversion of glycerol in *Ustilago maydis***. *PLoS One* 2011, **6**(1):e16438.

2. Liu Y, Koh CM, Sun L, Hlaing MM, Du M, Peng N, Ji L: **Characterization of glyceraldehyde-3-phosphate dehydrogenase gene Rt*GPD1* and development of genetic transformation method by dominant selection in oleaginous yeast *Rhodosporidium toruloides***. *Appl Microbiol Biotechnol* 2013, **97**(2):719-729.

3. Liu Y, Koh CM, Ngoh ST, Ji L: **Engineering an efficient and tight D-amino acid-inducible gene expression system in *Rhodosporidium/Rhodotorula* species**. *Microb Cell Fact* 2015, **14**(1):170.

4. Liu Y, Yap SA, Koh CM, Ji L: **Developing a set of strong intronic promoters for robust metabolic engineering in oleaginous *Rhodotorula* (*Rhodosporidium*) yeast species**. *Microb Cell Fact* 2016, **15**(1):200.
